# SPREAD, a framework and open-source R toolbox to quantify behavioural contagion

**DOI:** 10.1038/s44271-026-00538-0

**Published:** 2026-10-08

**Authors:** Jana Straßheim, Christophe Bousquet, Johanna Köchling, Britta Renner, Harald T. Schupp

**Affiliations:** 1 Department of Psychology, University of Konstanz, Universitätsstraße 10, Konstanz, Germany; 2 Centre for the Advanced Study of Collective Behaviour, University of Konstanz, Universitätsstraße 10, Konstanz, Germany

**Keywords:** Human behaviour, Human behaviour

## Abstract

Behavioural contagion, when one individual’s behaviour increases the likelihood of similar behaviours in others, is a fundamental dimension of social interaction, yet its empirical quantification remains methodologically challenging. Distinguishing contagion from coincidental co-occurrence, resolving contagion across continuous latencies rather than within fixed windows, accounting for behavioural base rates, and incorporating social structure require integrated analytical solutions. We present SPREAD, a conceptual and statistical framework implemented as an open-source R toolbox for quantifying behavioural contagion in time-series interaction data. SPREAD compares observed behavioural sequences with null distributions generated from permuted data that preserve individual event frequencies while disrupting inter-individual temporal alignment. The framework yields latency-resolved, base-rate corrected estimates of contagion strength that are comparable across behaviours and social configurations. We demonstrate SPREAD across two behavioural domains, laughter and eating, using human triadic data and illustrate its ability to detect contagion effects across distinct temporal dynamics.

## Abstract (web summary)

The SPREAD framework provides a conceptual and statistical framework to quantify behavioural contagion. It is implemented as an open-source R toolbox.

## Introduction

Human behaviour is deeply shaped by the social environment in which it occurs^1^. One particularly striking phenomenon is behavioural contagion, referring to the spontaneous spread of behaviours from one person to another within a temporal window ranging from milliseconds to minutes. Across the literature, contagion has been conceptualised in multiple ways, ranging from reflex-like mimicry to temporally extended forms of behavioural contagion, including affective convergence^2,3^. Classic examples include yawning, laughter, and smiling, rapid expressive behaviours that can be triggered almost reflexively upon observation^4,5^. The same contagion logic can also be applied to more temporally extended and structured behaviours such as eating, where contagion manifests in coordinated bite timing and matched food choices within social interaction^6,7^.

Behavioural contagion is embedded in social structure. Both social relationships and group size shape how behaviours spread, influencing not only the likelihood of transmission but also the form that contagion may take^2,3^. Who interacts with whom, and how many individuals are present, determine the relational pathways through which behavioural influence unfolds. From a methodological perspective, this implies that contagion should often go beyond the dyadic level and be examined within the broader relational and structural context in which behaviour occurs.

In addition, behavioural contagion is inherently dynamic: responses emerge at specific latencies and may accumulate across multiple individuals over the course of an interaction. Such temporal alignment can give rise to behavioural synchrony, reinforcing social cohesion and positive affect among group members^8–10^. This temporal perspective aligns with work in social neuroscience, particularly the second-person neuroscience framework^11,12^, which emphasises the importance of studying behaviour as it unfolds within ongoing, reciprocal social interactions.

Despite growing theoretical interest, the empirical quantification of behavioural contagion remains heterogeneous. Studies differ substantially in how contagion is operationalised, how temporal windows are defined, whether behavioural base rates are controlled, and how social configuration is incorporated^2,10,13,14^. Existing approaches include time-lagged analyses that test predictive dependencies within a (pre-)defined temporal window^15,16^ and permutation-based methods that compare observed temporal co-occurrence against chance expectations^6,17^. Although recent theoretical accounts emphasise the conceptual complexity and multi-level nature of contagion processes^2,3^, methodological tools capable of operationalising these distinctions remain limited and no unified framework currently integrates these challenges within a single statistical architecture capable of resolving contagion dynamics across time and social configurations.

A primary challenge is distinguishing genuine contagion from coincidental co-occurrences arising by chance^14^. Establishing contagion requires analytical approaches that move beyond simple temporal alignment. To this end, we apply time-series permutations of observed and randomised data to quantify the extent to which behavioural alignment exceeds chance expectations.

A second challenge concerns the temporal resolution of contagion processes. Contagion unfolds across time and may vary in latency depending on the behavioural domain and interaction context^4,15,16,18^. Approaches that rely on predefined or fixed time windows risk masking this variability. Methods that quantify contagion across a range of time resolutions can instead reveal the dynamics and richness of contagious effects without imposing arbitrary temporal boundaries.

A third challenge relates to behavioural base rates. Individuals differ in their baseline frequency of producing specific behaviours, and these base rates may fluctuate across contexts or over time^13^. Frequently occurring behaviours are more likely to coincide by chance, which can inflate estimates of contagion if not properly controlled^14^. Robust quantification of behavioural contagion therefore requires analytical approaches that preserve individual event frequencies, effectively controlling for inter-individual differences in base rate^6,17^.

As contagion does not occur in isolation but within structured social systems, a fourth challenge concerns social configuration, variation in group size, relational structure and directional influence^2,3^. Importantly, intragroup convergence cannot be reduced to a simple aggregation of dyadic effects, but may involve emergent higher-order dynamics^3^. In dyadic interactions, contagion can be described in terms of whether a response occurs at a given latency. As group size increases, additional and qualitatively distinct contagion effects may emerge. In triads, for example, an individual may influence, or be influenced by, one partner (a dyadic effect) or both partners (a triadic effect). More generally, contagion strength may scale with the number or proportion of group members expressing a behaviour, and may vary depending on leader-follower dynamics or preferential sensitivity to specific individuals^19,20^. Analytical tools must therefore resolve two complementary aspects of social configuration: they must distinguish dyadic from higher-order contagion effects by accommodating variation in group size and relational structure, and they must capture the directionality of influence by supporting both forward-looking analyses, which adopt a leader perspective and quantify the influence exerted by a focal individual on subsequent behaviour in others, and backward-looking analyses, which adopt a follower perspective and quantify the influence of prior behaviour by others on the focal individual.

These challenges highlight that although permutation-based methods have been applied to specific aspects of contagion detection, they have typically been implemented as context-specific tests rather than integrated into a unified framework.

**Fig. 1:**
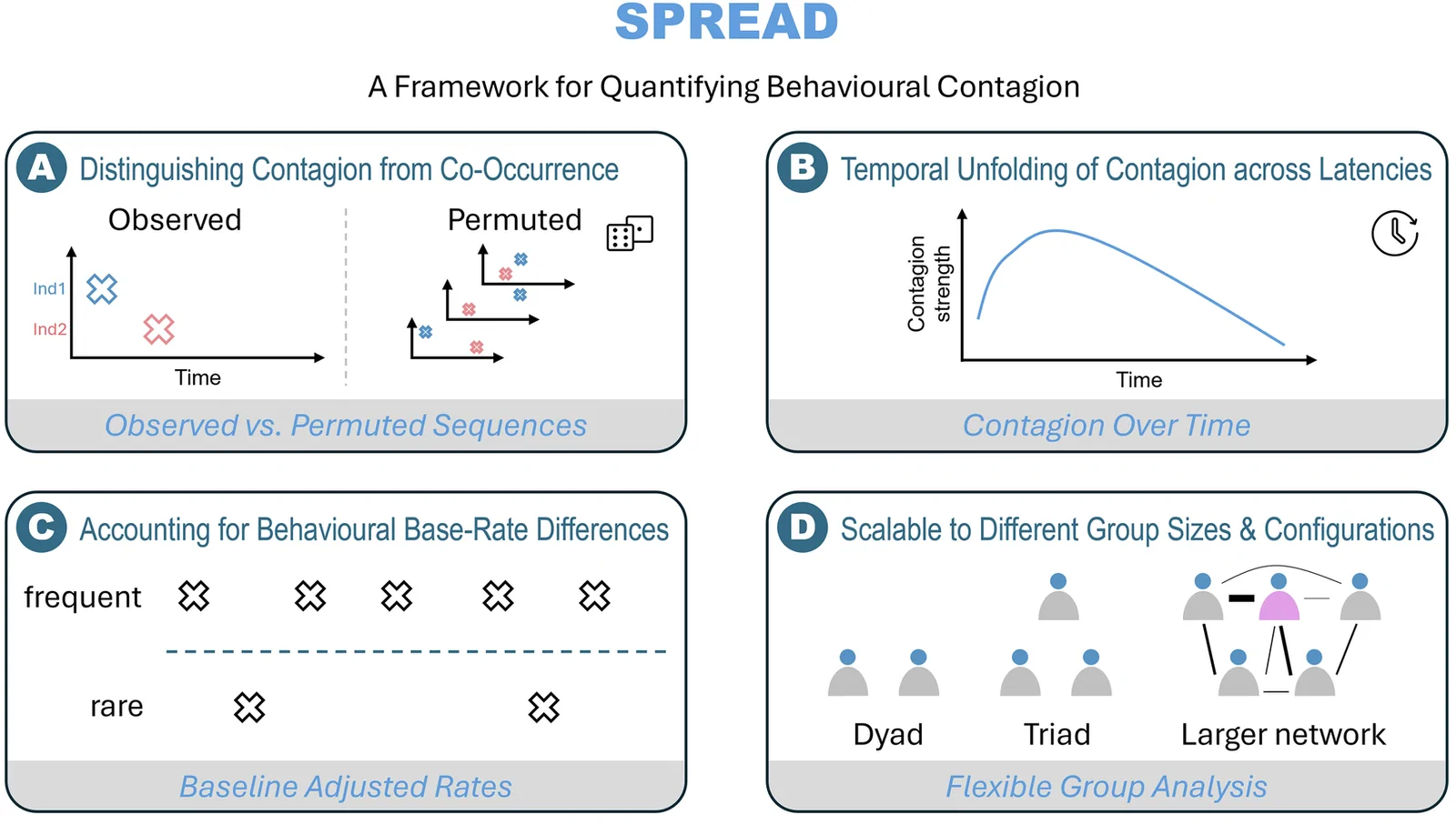
Overview of the four conceptual dimensions underlying the SPREAD framework for assessing behavioural contagion. SPREAD distinguishes behavioural contagion from coincidental temporal overlap by comparing observed and permuted sequences (**A**), resolves contagion strength across latencies (**B**), and adjusts for behavioural base-rate differences (**C**). In addition, SPREAD is scalable across group sizes and social configurations, enabling analyses from dyads to larger social networks (**D**).

Here, we introduce SPREAD, a conceptual and statistical framework implemented as an open-source R toolbox for quantifying behavioural contagion in time-series interaction data. Figure 1 summarises the four dimensions that form the conceptual basis of the SPREAD framework. Statistically, SPREAD compares observed behavioural sequences against null distributions generated from permuted data. The framework thereby yields latency-resolved, base-rate-corrected estimates of contagion strength that can be compared across behaviours, group sizes, and social configurations, from dyads to larger social networks. We validate SPREAD using data from real-world social interactions involving three individuals (triads) across two behavioural domains: laughter, a rapid and primarily socially driven behaviour, and eating, a slower behaviour shaped by both biological regulation and social context, operating on distinct temporal scales. These domains place distinct demands on temporal resolution, baseline correction, and sensitivity to social structure, including both dyadic and triadic effects.

## Methods and data

### Statistical architecture of the SPREAD framework

SPREAD formalises behavioural contagion detection as a permutation-based, latency-resolved estimation problem in event-based time series. Rather than testing temporal overlap within predefined windows, the framework compares observed behavioural event sequences against null distributions that preserve individual event frequencies while disrupting inter-individual temporal alignment.

The analytical architecture consists of three integrated steps. First, behavioural events are represented as discrete time-stamped occurrences, allowing contagion strength to be estimated as a continuous function of lag relative to a focal event. Second, null models are generated by randomly permuting event timestamps within the observed interaction window for each individual. Third, observed and permuted contagion profiles are compared using standardised statistics to quantify the magnitude and direction of behavioural contagion across latencies.

**Fig. 2:**
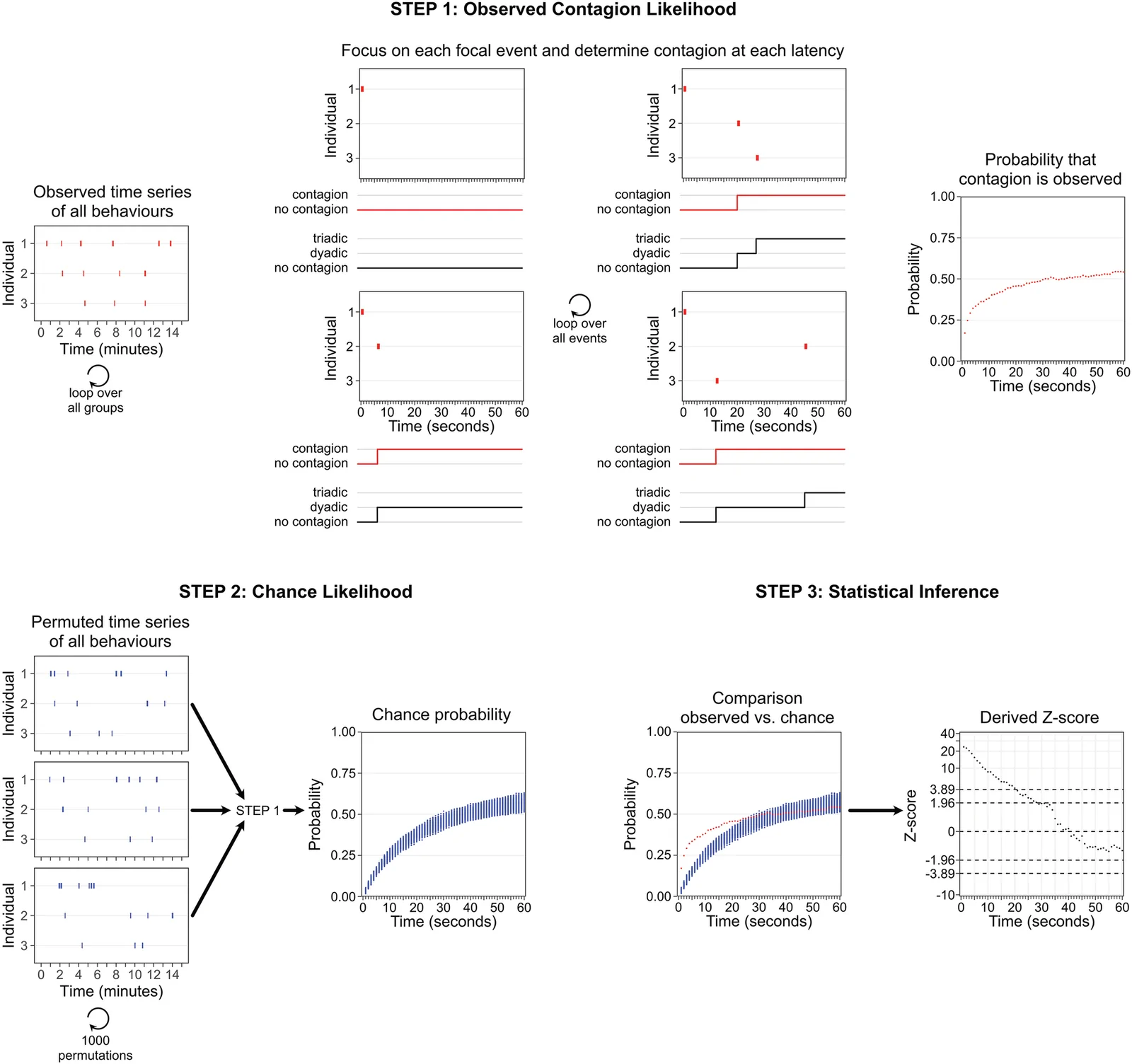
Overview of the statistical SPREAD framework for quantifying behavioural contagion across latencies and social configurations. SPREAD quantifies behavioural contagion using a three-step permutation-based procedure operating at two analytical levels: a coarse-grained assessment of contagion versus no contagion, and a finer-grained decomposition of contagion within groups (e.g., dyadic and triadic effects in a group of three). Red dots represent observed probabilities derived from the empirical data, and blue dots represent the corresponding null distributions obtained from permuted data. Step 1 (Observed Contagion Likelihood): For each focal event, the procedure determines whether the same behaviour occurs in other group members across increasing latency thresholds. The analysis is applied independently to each group (“loop over all groups”) and aggregated across groups to yield latency-resolved probabilities of behavioural co-occurrence. Events are further stratified to produce separate observed profiles for dyadic and triadic effects. Step 2 (Chance Likelihood): Individual time-series are randomly permuted (1000 iterations) to retain each individual’s event frequency while randomising inter-individual temporal alignment. Reapplying Step 1 to the permuted data yields null distributions of expected co-occurrence probabilities across latencies for overall, dyadic, and triadic effects. Step 3 (Statistical Inference): At each latency, observed contagion probabilities (red) are compared with their corresponding null distributions (blue), and the difference is standardised as a Z-score to quantify effect magnitude and direction. This enables inference of facilitation, inhibition, or absence of social effects without reliance on predefined temporal windows.

The workflow is implemented in R version 4.5.2^21^ and schematically illustrated in Fig. 2.

### Estimation of observed contagion likelihood from event-based time series

In the first analytical step, onset times are extracted for each behavioural event and individual, yielding event-based time series within a defined interaction window. Each event is treated as a focal reference point from which temporal lags to events produced by other group members are computed.

Rather than relying on a single predefined temporal window, SPREAD estimates observed contagion likelihood as a function of lag across a predefined latency range. This range is operationalised by progressively expanding the maximum lag threshold of the latency interval and recalculating contagion likelihood at each increment. In the present analyses, latency was evaluated from 1 to 60 seconds in 1-second increments.

For each focal behavioural event and latency threshold, SPREAD quantifies whether the same behaviour is initiated by other group members within the specified lag interval. At a coarse-grained level, observed contagion is defined as the probability that at least one other individual engages in the behaviour within the latency threshold. At a finer-grained level, responses are decomposed according to the number of responding group members.

For each latency, probabilities are computed by aggregating across focal events, individuals, and groups, yielding an observed contagion profile as a continuous function of lag.

### Estimation of chance contagion likelihood via permutation-based null models

To distinguish behavioural contagion from coincidental temporal co-occurrence, SPREAD generates permutation-based null models. For each individual, observed event timestamps are randomly permuted within the original interaction interval while maintaining the total number of events and the overall interaction duration. Permutations are performed independently for each individual, so that individual event frequencies are retained while their temporal co-occurrence is randomised.

For each permuted dataset, the same latency-resolved estimation procedure described above is applied, yielding a distribution of chance-level contagion likelihood across latencies. The permutation procedure is repeated across a large number of iterations (e.g., 1,000 in the present analyses), producing empirical null distributions of contagion strength at each latency threshold. Analyses using 10,000 permutations yielded nearly identical results, indicating stability with respect to the number of permutation iterations (Supplementary Fig. 2 and 3).

### Statistical inference and effect-size estimation

For each latency threshold, observed contagion strength is compared to the corresponding permutation-based null distribution. Deviation from chance expectations is quantified by standardising the observed estimate relative to the mean and standard deviation of the permutation-based distribution. This standardisation yields latency-resolved measures of contagion strength that reflect both magnitude and direction. Positive values indicate facilitative contagion, whereas negative values indicate inhibitory effects. In addition to Z-scores, SPREAD reports the Incidence Rate Ratio (IRR) as a complementary effect-size measure^22^. The IRR expresses the multiplicative difference between observed and permutation-based event rates at a given latency. An IRR greater than 1 indicates that temporal co-occurrence exceeds chance expectations, whereas an IRR below 1 indicates reduced co-occurrence relative to the null model. Confidence intervals for the IRR are derived from the empirical permutation-based distribution.

The inferential approach characterises behavioural contagion as a latency-resolved profile evaluated against permutation-derived null distributions. Statistical thresholds use two-sided standard-normal criteria applied to Z-scores at each cumulative latency threshold. Inference is pointwise rather than omnibus across the full latency profile. Accordingly, no family-wise adjustment is applied across latencies^23^.

The mathematical specification is provided in the Mathematical formulation section below.

### Modelling social structure and directionality

Beyond estimating overall contagion likelihood, the framework enables the analysis of contagion effects according to the number of responding group members. In triadic datasets, this distinguishes dyadic effects (one responder) from triadic effects (two responders). This logic generalises to larger groups by extending response counts to higher-order configurations, for example by considering the number or proportion of group members responding after a focal event.

In addition to group size, SPREAD allows contagion analyses to be conditioned on social roles or relational categories (e.g., familiar versus unfamiliar partners, asymmetric interaction roles, or leader-follower structures). By preserving the full relational configuration of the group, the framework enables examination of whether contagion varies as a function of interaction asymmetry or exposure patterns.

Directionality of influence can be examined through both forward-looking and backward-looking analyses within the same statistical architecture. In forward-looking analyses, the focal individual is treated as the emitter, and the analysis estimates whether the focal individual’s behaviour was followed by behaviour in one or more group members. This perspective is adopted in the case studies presented in the Validation section. In backward-looking analyses, the focal individual is treated as the potential receiver, and the analysis estimates whether the focal individual’s behaviour was preceded by behaviour in one or more group members. These complementary perspectives allow SPREAD to assess contagion from both emitter and receiver perspectives.

### Control analyses

To examine the statistical specificity of the SPREAD framework, we conducted control analyses designed to assess whether detected contagion effects could arise from artefactual temporal structure, random co-occurrence, or within-individual temporal dependence rather than genuine inter-individual contagion effects. These analyses test whether the framework selectively detects structured contagion while remaining insensitive to non-social temporal patterns.

#### Constructed group controls

To assess the false-positive rate of the framework, artificial groups were constructed by combining individuals drawn from different original interaction groups. Because these individuals did not share a common interaction context, genuine inter-individual contagion is absent by design.

The SPREAD procedure described above was applied to these constructed groups without modification. This analysis tests whether the permutation-based architecture falsely detects contagion in the absence of shared interaction dynamics. Under appropriate specificity, observed contagion likelihood in constructed groups should not systematically exceed chance expectations derived from the permutation-based null model.

#### Within-individual temporal dependence

To distinguish inter-individual contagion from intra-individual repetition tendencies, we estimated latency-resolved within-individual temporal dependence within each individual’s behavioural stream. This control analysis tested whether an individual’s own behaviour increased the likelihood of immediate repetition, for example through short-term behavioural carry-over effects, habitual repetition, or other self-reinforcing dynamics.

We used the same latency windows and estimation procedure as in the contagion analyses, but redefined coincidence as occurring within individuals rather than across group members. This control assesses whether short-latency clustering reflects self-triggered repetition rather than socially structured contagion.

### Mathematical formulation

The SPREAD framework can be formally expressed as follows:

Let $G=\{{g}_{1},...,{g}_{N}\}$ denote the set of groups. Each group g ∈ G consists of Mg individuals observed over a time interval [0,T].

For each individual *i* ∈ *g*, let

$${E}_{i}=\{{t}_{i,1},...,{t}_{i,{n}_{i}}\}\subset [0,T]$$

denote the ordered onset times of behavioural events.

Let $L=\{{l}_{1},...,{l}_{L}\}$ be the set of latency thresholds.

#### Observed coincidence structure

For each focal event ${t}_{i,k}$ ∈ ${E}_{i}$ and latency l∈L, define the latency interval

$${W}_{i,k}(l)=({t}_{i,k},{t}_{i,k}+l].$$

For each individual j∈g (including *j* = *i*), define the pairwise coincidence indicator:

$${C}_{\left(i,j,k\right)}(l) = \left\{\begin{array}{ll}1&{{if}\,\exists {t}_{j,m}\in {E}_{j}{{\rm{\backslash }}}\left\{{t}_{i,k}\right\}{such}\,{that}\,{t}_{j,m}\in {W}_{i,k}(l)} \\ 0&{{otherwise}}\hfill \end{array}\right.$$

For each focal event, we define two derived metrics at each latency *l*:

- Within-individual repetition:
  ${C}_{{egocentric}}(i,k,l)={C}_{i,i,k,l}$, equalling 1 if *i* repeats the behaviour within latency *l*
- How many other individuals showed the behaviour within latency *l*:

$${M}_{{others}}(i,k,l)={\sum}_{j\in g,j\ne i}{C}_{i,j,k,l}$$

We can then define five categories depending on the relationship between these metrics:

- $I({C}_{{egocentric}}=0\wedge {M}_{{ot}h{ers}}=0)$ when no contagion occurred
- $I({C}_{{egocentric}}=1\wedge {M}_{{ot}h{ers}}=0)$ when only focal individual repeats the behaviour
- $I({C}_{{egocentric}}=0\wedge {M}_{{ot}h{ers}}=1)$ when only one other individual shows the behaviour
- $I({C}_{{egocentric}}=0\wedge {M}_{{ot}h{ers}}=2)$ when both other individuals show the behaviour
- $I({C}_{{egocentric}}=0\wedge {M}_{{ot}h{ers}}\ge 1)$ when one or both other individuals show the behaviour

The total observed coincidence counts for each category at latency *l* are, after summing across groups, individuals and events:

- $${S}_{{obs}}^{{no}\,{contagion}}(l)={\sum }_{g\in G}{\sum }_{i\in g}{\sum }_{k=1}^{{n}_{i}}I({C}_{{egocentric}}=0\wedge {M}_{{others}}=0)$$
- $${S}_{{obs}}^{{egocentric}}(l)={\sum }_{g\in G}{\sum }_{i\in g}\mathop{\sum }_{k=1}^{{n}_{i}}I({C}_{{egocentric}}=1\wedge {M}_{{others}}=0)$$
- $${S}_{{obs}}^{{dyadic}}(l)={\sum }_{g\in G}{\sum }_{i\in g}\mathop{\sum }_{k=1}^{{n}_{i}}I({C}_{{egocentric}}=0\wedge {M}_{{others}}=1)$$
- $${S}_{{obs}}^{{triadic}}(l)={\sum }_{g\in G}{\sum }_{i\in g}\mathop{\sum }_{k=1}^{{n}_{i}}I({C}_{{egocentric}}=0\wedge {M}_{{others}}=2)$$
- $${S}_{{obs}}^{{overall}}(l)={\sum }_{g\in G}{\sum }_{i\in g}\mathop{\sum }_{k=1}^{{n}_{i}}I({C}_{{egocentric}}=0\wedge {M}_{{others}}\ge 1)$$

Note that ${S}_{{obs}}^{{overall}}(l)={S}_{{obs}}^{{dyadic}}(l)+{S}_{{obs}}^{{triadic}}(l)$ in our case.

Therefore, for each category *c*, we have an associated observed count ${S}_{{obs}}^{(c)}(l)$.

#### Null model

Under the permutation-based null model, event timestamps are randomly reassigned within $\left[0,T\right]$ independently for each individual *i*, preserving ${n}_{i}$.

For each replicate r=1,...,R, this yields permuted event sets ${E}_{i}^{(r)}$ and corresponding coincidence counts ${S}_{{perm}}^{(c)}(l,r)$ for each category *c*. This permutation approach allows for the generation of an empirical null distribution without relying on parametric assumptions^24^.

The empirical null distribution for category *c* at latency *l* is:

$$\left\{{S}_{{perm}}^{(c)(1)}(l),...,{S}_{{perm}}^{(c)(R)}(l)\right\}$$

with mean:

$${\mu }_{{perm}}^{(c)}(l)=\frac{1}{R}\mathop{\sum }_{r=1}^{R}{S}_{{perm}}^{(c)}(l,r)$$

and standard deviation:

$${\sigma }_{{perm}}^{\left(c\right)}\left(l\right)={sd}\left({\left\{{S}_{{perm}}^{\left(c\right)}\left(l,r\right)\right\}}_{r=1}^{R}\right)$$

#### Standardised contagion strength

The latency-resolved Z-score for each category *c* is defined as:

$${Z}^{\left(c\right)}\left(l\right)=\frac{{S}_{{obs}}^{\left(c\right)}\left(l\right)-{\mu }_{{perm}}^{\left(c\right)}\left(l\right)}{{\sigma }_{{perm}}^{\left(c\right)}\left(l\right)}$$

#### Incidence rate ratio (IRR)

The Incidence Rate Ratio (IRR^22^) provides a complementary multiplicative effect-size measure defined as

$${{IRR}}_{{obs}}^{(c)}(l)=\frac{{S}_{{obs}}^{(c)}(l)}{{\mu }_{{perm}}^{(c)}(l)}$$

An ${{IRR}}^{(c)}(l) > 1$ indicates that the observed coincidence count exceeds the expected count under the permutation-based null model, whereas ${{IRR}}^{(c)}(l) < 1$ indicates lower-than-expected temporal co-occurrence. It can be interpreted as the multiplicative factor between the observed and the permuted counts: a value of 2 indicates that the observed count is twice as high as the permuted count and a value of 0.5 indicates that the observed count is twice as low as the permuted count.

To quantify uncertainty, a permutation-based confidence interval is derived using the percentile method^25^. For each permutation replicate r=1,...,R, a ratio is computed as:

$${{IRR}}_{{perm}}^{(c)}\left(l,r\right)=\frac{{S}_{{perm}}^{(c)}(l,r)}{{\mu }_{{perm}}^{(c)}(l,r)}$$

The 95% confidence interval (CI) for ${{IRR}}^{(c)}(l)$ is defined by the 2.5th and 97.5th percentiles of the empirical distribution $\left[\frac{{{IRR}}_{{obs}}^{(c)}(l)}{{Q}_{0.975}},\frac{{{IRR}}_{{obs}}^{(c)}(l)}{{Q}_{0.025}}\right]$.

In cases where the permutation-based expected count ${\mu }_{{perm}}^{(c)}(l)$ is very small, leading to unstable upper bounds, values exceeding a predefined reporting threshold (1000) are truncated for interpretability.

#### Normalisation for visual representation

For visualisation purposes, we express the results as a percentage of the total opportunity for contagion. Therefore, the observed and permuted counts are normalised by the total number of events in the dataset at each latency *l*:

$${p}_{{obs}}^{(c)}\left(l\right)=\frac{{S}_{{obs}}^{(c)}\left(l\right)}{{N}_{{total}}(l)}$$

$${p}_{{perm}}^{(c)}\left(l,r\right)=\frac{{S}_{{perm}}^{(c)}\left(l,r\right)}{{N}_{{total}}(l)}$$

where ${N}_{{total}}(l)$ is the count of focal events ${t}_{i,k}$ such that the latency window ${W}_{i,k}(l)$ is fully contained within the observation interval $\left[0,T\right]$.

This normalisation maps the counts to a scale of 0 to 1 (or 0% to 100%), allowing the contagion likelihood to be plotted on an intuitively interpretable axis. However, the Z-scores and IRRs remain derived from the unnormalized counts (${S}_{{obs}}^{(c)}(l)$ and ${S}_{{perm}}^{(c)}(l,r)$), ensuring that the statistical inference is unaffected by this scaling.

### Demonstration datasets

To provide empirical contexts for demonstrating the applicability and scalability of SPREAD, we applied the framework to two independent datasets derived from social interactions. The selected cases differ in regulatory structure, and expected temporal resolution, thereby providing complementary contexts for evaluating the framework’s ability to resolve latency-specific contagion patterns. Neither the original studies nor the present analyses were preregistered; these datasets serve as empirical demonstrations of the statistical architecture rather than tests of preregistered behavioural hypotheses.

#### Case 1: Laughter in triadic social interaction

The first dataset was derived from a previously conducted experimental study of triadic social interaction (see^26^ for full experimental details). For the present study, laughter events were newly extracted and systematically coded from the original audio recordings to demonstrate the SPREAD framework.

Triads discussed either relaxed or tense topics under two conditions: social eating or discussion in the absence of food. Each conversation lasted approximately 15 min. The sample included 84 participants forming 28 triads. Gender was self-reported: 58 participants (69%) identified as female, 26 (31%) as male, and none identified as diverse. The mean age was 24.0 years (SD = 7.4, range = 19-61). Data on race or ethnicity were not collected. The study was approved by the ethics committee of the University of Konstanz (No. 24/2020) and conducted in accordance with the Declaration of Helsinki. All participants provided written informed consent prior to participation. Participants received a lunch meal or a €10 food voucher as compensation, and psychology students received an additional 0.5 hours of course credit.

Social interactions were recorded via Zoom (Version 5.6.5). Audio files from each participant were processed to extract laughter events. We used Audacity (https://www.audacityteam.org/) and its “label sounds” function to detect events with the following parameters: threshold = −30.0 dB, measurement = RMS level, minimum silence = 0.2 s, minimum label interval = 0.8 s, maximum leading silence = 0 s, and maximum trailing silence = 0 s. Background noise was removed, and each segment was classified as laughter, speech, or filler (e.g., sighs, hums). When speech and laughter co-occurred, the event was classified as speech; when laughter co-occurred with filler sounds, it was classified as laughter. All detected sounds were manually reviewed to ensure accuracy. Laughter onset times were extracted, and the resulting time-series data were analysed using SPREAD.

Overall, participants produced 1,032 laughter events. For the forward-looking analysis in observed triads, one event was excluded because it occurred within 1 s of the end of the interaction, and therefore could not be followed by a potential response within the shortest latency window. This resulted in 1,031 observed laughter events available for estimating contagion in observed triads. For the constructed-triad control analysis, the effective duration of each artificial triad was defined by the shortest interaction duration among the three observed triads from which it was assembled. Consequently, laughter events occurring after this time point in the two remaining observed triads were excluded. This resulted in 989 laughter events available for estimating contagion in constructed triads.

#### Case 2: Eating behaviour during triadic interaction

The second dataset examined eating behaviour in triads engaged in spontaneous conversation while eating. Each conversation lasted approximately 15 min. This condition formed part of a broader experimental design that also included no-food control conditions and cooperative tasks. The study was conducted in-person in the laboratory between 2022 and 2023 and included 36 triads (108 participants) in the eating condition. Gender was self-reported: 76 participants (70.4%) identified as female, 31 male (28.7%), and one identified as diverse (0.9%). The mean age was 21.8 years (SD = 3.1, range = 18-37). Data on race or ethnicity were not collected. The study was approved by the ethics committee of the University of Konstanz (No. 3/2022) and conducted in accordance with the Declaration of Helsinki. All participants provided written informed consent prior to participation. Participants received €20 or two hours of course credit as compensation.

The eating condition was standardised: all participants received identical plates containing a slice of cake and a fork positioned in fixed locations. Body movements were recorded using motion-capture sensors attached prior to the session, and interactions were video-recorded from four camera angles. Eating-related behaviours were coded from the camera providing the clearest view using a custom ethogram comprising five categories: touching the fork, holding the fork, collecting food on the plate, transporting food to the mouth, and biting the food. Additional codes included plate rotation, plate empty, and experimenter reminders to eat.

For the present methodological application of SPREAD, we focused on bite events as the behaviours most directly indexing eating. Bite onset times were defined as the video frame in which the fork entered the mouth and were extracted as discrete event timestamps for analysis using SPREAD.

Overall, participants produced 1,427 bite events. For the forward-looking analysis in observed triads, one event was excluded because it occurred within 1 s of the end of the interaction and therefore could not be followed by a potential response within the shortest latency window. This resulted in 1,426 bite events available for estimating eating contagion in observed triads.

For the constructed-triad control analysis, the effective duration of each artificial triad was defined by the shortest interaction duration among the three observed triads from which it was assembled. Consequently, bite events occurring after this time point in the remaining two triads were excluded. This resulted in 1,411 bite events available for estimating eating contagion in constructed triads.

## Validation

To empirically assess the robustness and specificity of SPREAD, we applied the framework to two behavioural domains that differ in social and temporal dynamics. Case 1 examines laughter, a rapid communicative behaviour occurring in brief episodes, whereas Case 2 examines eating, a behaviour unfolding over longer timescales. Together, the two cases place complementary demands on latency resolution, base-rate correction, and sensitivity to social configuration. This comparative application allows us to assess whether SPREAD consistently detects latency-resolved deviations from chance across qualitatively distinct behavioural dynamics.

### Case 1: Laughter in triadic interaction

**Fig. 3:**
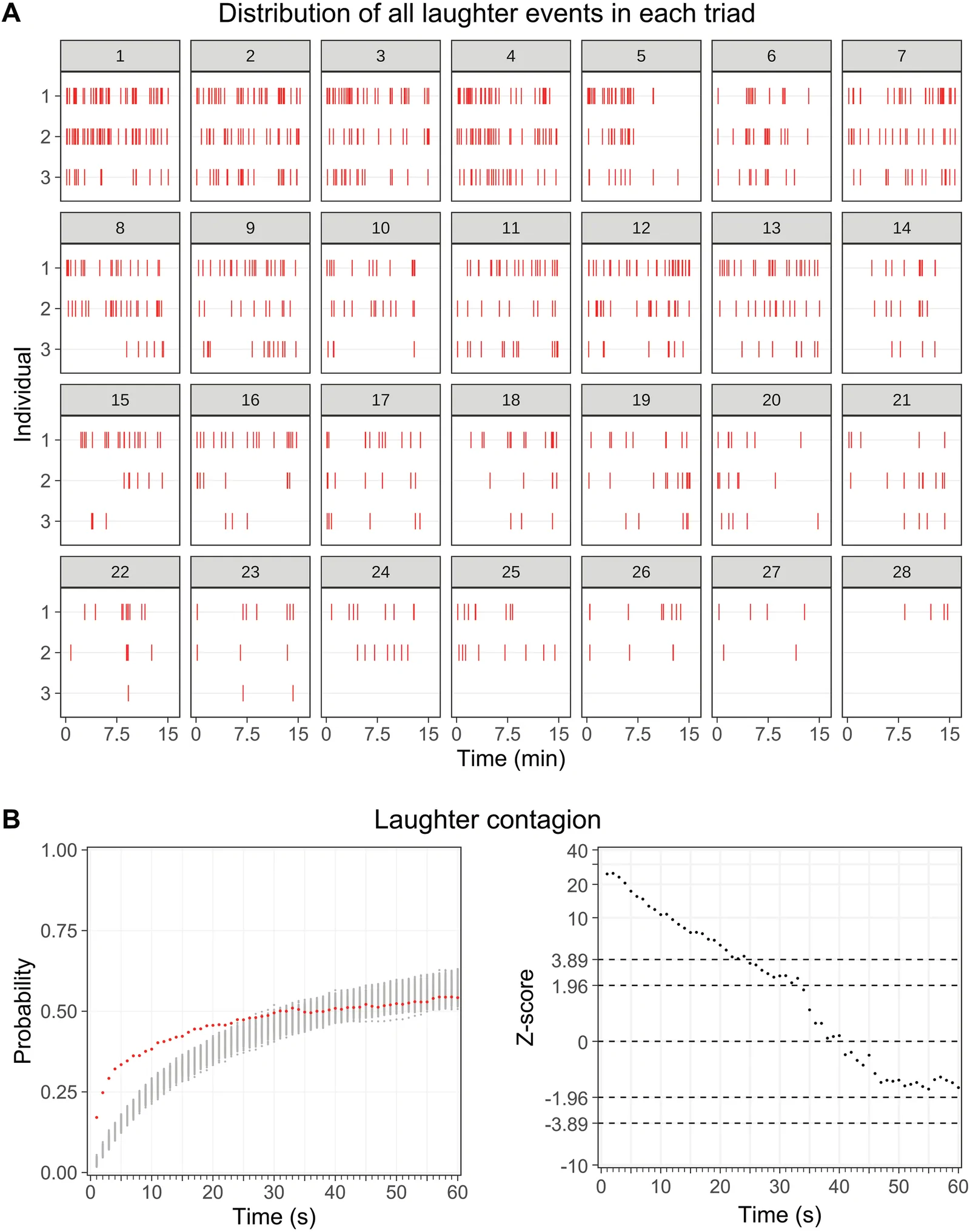
Case 1: Laughter contagion. **A** Distribution of laughter events for each triad (*N* = 1032) across the approximately 15-minute conversation. Triads (arranged from top left to bottom right) and individuals within each triad (1–3) are ranked by total laughter count. Short laughter events (≤ 3 s) are slightly expanded to improve visualisation. **B** Left: Observed (red dots) and permuted (grey dots, 1000 permutations) probabilities of laughter contagion as a function of latency. **B** Right: Latency-resolved Z-scores quantifying the deviation of observed probabilities from chance. |Z | ≥ 1.96 indicates *p* ≤ 0.05 and |Z | ≥ 3.89 indicates *p* ≤ 0.0001. **B** is based on N = 1031 laughter events. One was excluded because it occurred within 1 s of the end of the interaction and could therefore not be evaluated for potential contagion. Statistical inference is based on pointwise, two-sided Z-tests using permutation-derived null distributions generated from 1000 permutations. Pointwise *p*-values for each cumulative latency threshold are reported in Table S1.

The dataset comprised 28 groups (N = 84 individuals) who engaged in approximately 15-minute conversations, yielding 1,032 laughter events. Laughter frequency varied substantially across groups (36.9 ± 25.4 events per group; range = 4–101) and across individuals (12.3 ± 10.0 per participant; range = 0–43). The distribution of laughter events across conversations is shown in Fig. 3A.

We then tested whether these laughter events reflected contagion rather than chance co-occurrence. Figure 3B, left panel shows the observed (red dots) and permuted (grey dots; 1,000 permutations) latency-resolved probability distributions. The observed probability that laughter by other group members followed a focal laughter event increased from 17.1% within the first second to 38.2% within 10 s, 45.6% within 20 s, and 49.5% within 30 s. Relative to the permutation-based null distributions, observed probabilities were markedly elevated at short latencies, particularly within the first 20 s.

Statistical inference confirmed this pattern (Fig. 3B, right panel; Table S1). Laughter contagion was already evident within the 0-1 s window and peaked in the 1-2 s window (IRR = 3.60 [95% CI: 3.02, 4.47], *Z* = 24.99, *p* < 0.0001). Effects remained significant up to 24 s (IRR = 1.14 [95% CI: 1.07, 1.22], *Z* = 4.21, *p* < 0.0001), and persisted with decreasing magnitude until approximately 33 s (IRR [95% CI] = 1.08 [1.01, 1.15], *Z* = 2.40, *p* < 0.05).

Further analyses examined contagion according to whether a focal laughter event was followed by one or by both of the other members of the triad.

**Fig. 4:**
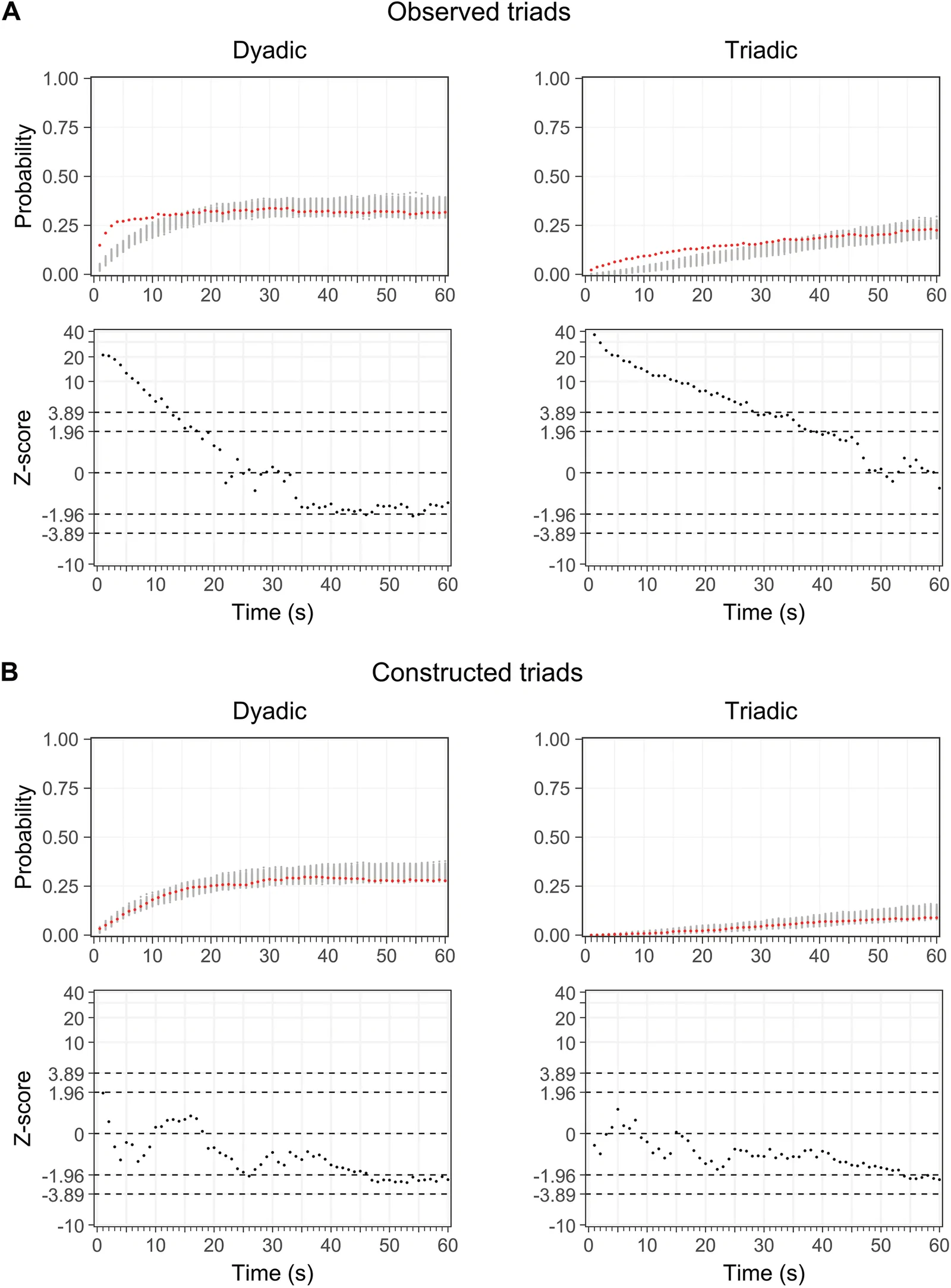
Case 1: Laughter contagion in observed and constructed triads. **A** Latency-resolved probabilities of contagion and corresponding Z-scores for observed triads, shown separately for dyadic (left) and triadic (right) effects. **B** Latency-resolved probabilities of contagion and corresponding Z-scores for constructed triads, shown separately for dyadic (left) and triadic (right) effects. In the probability panels (top row), red dots represent observed data and grey dots represent permuted data. |Z | ≥ 1.96 indicates *p* ≤ 0.05 and |Z | ≥ 3.89 indicates *p* ≤ 0.0001. Analyses are based on *N* = 1031 laughter events for observed triads and *N* = 989 laughter events for constructed triads. Statistical inference is based on pointwise, two-sided Z-tests using permutation-derived null distributions generated from 1000 permutations. Pointwise p-values for each cumulative latency threshold are reported in Tables S2–S5.

When laughter spread to one individual (dyadic effects; Fig. 4A, left panels; Table S2), contagion was detectable in the 0–1 s window: compared with 3.46% expected under the null model, laughter occurred with a probability of 14.84% (IRR [95% CI] = 4.29 [3.26, 6.12], *Z* = 21.01, *p* < 0.0001). Dyadic effects remained significant up to 12 s (30.36% observed vs. 25.17% expected; IRR [95% CI] = 1.21 [1.11, 1.31], *Z* = 4.61, *p* < 0.0001) and persisted with decreasing magnitude until 17 s (31.58% vs. 29.15%; IRR [95% CI] = 1.10 [1.00, 1.18], *Z* = 2.00, *p* < 0.05).

When laughter spread to both other individuals (triadic effects; Fig. 4A, right panels; Table S3), effects were stronger at short latencies and persisted over a longer temporal window. Triadic contagion emerged within the first second (2.23% vs. 0.035%; IRR [95% CI] = 62.68 [11.50, >1,000], *Z* = 36.5, *p* < 0.0001), remained significant up to 28 s (15.27% vs. 11.20%; IRR [95% CI] = 1.36 [1.14, 1.64], *Z* = 4.04, *p* < 0.0001), and persisted with reduced magnitude until 38 s (17.93% vs. 15.61%; IRR [95% CI] = 1.15 [1.00, 1.35], *Z* = 1.96, *p *< 0.05).

Across latencies, contagion strength and temporal persistence were greater when both group members showed the behaviour than when only one did, consistent with contagion dynamics scaling with the number of responding individuals.

In contrast to observed triads, constructed triads showed no evidence of sustained contagion at either the dyadic or triadic level across latencies (Fig. 4B; Tables S4 and S5). Z-scores remained close to zero across latencies and did not exceed conventional significance thresholds, including within the temporal windows in which robust contagion effects were observed in real triads. Together, these results suggest that the temporally extended laughter contagion observed in real social groups is unlikely to be attributable solely to chance co-occurrence or baseline event structure. Moreover, the graded pattern observed in real triads, with stronger and more persistent effects when both group members responded, was absent in constructed triads.

To further rule out within-individual temporal dependencies as an alternative explanation, we conducted an additional specificity check assessing whether the dyadic and triadic effects could reflect within-individual repetition rather than inter-individual contagion (Supplementary Fig. 1, left column). Results showed the opposite pattern. For laughter, within-individual repetition probabilities were significantly lower than expected under the permutation-based null model across latencies from 1 to 60 s (all IRRs ≤ 0.77, -5.28 ≤ Z ≤ -2.55, *p* ≤ 0.05; Table S11).

### Case 2: Eating behaviour in triadic interaction

**Fig. 5:**
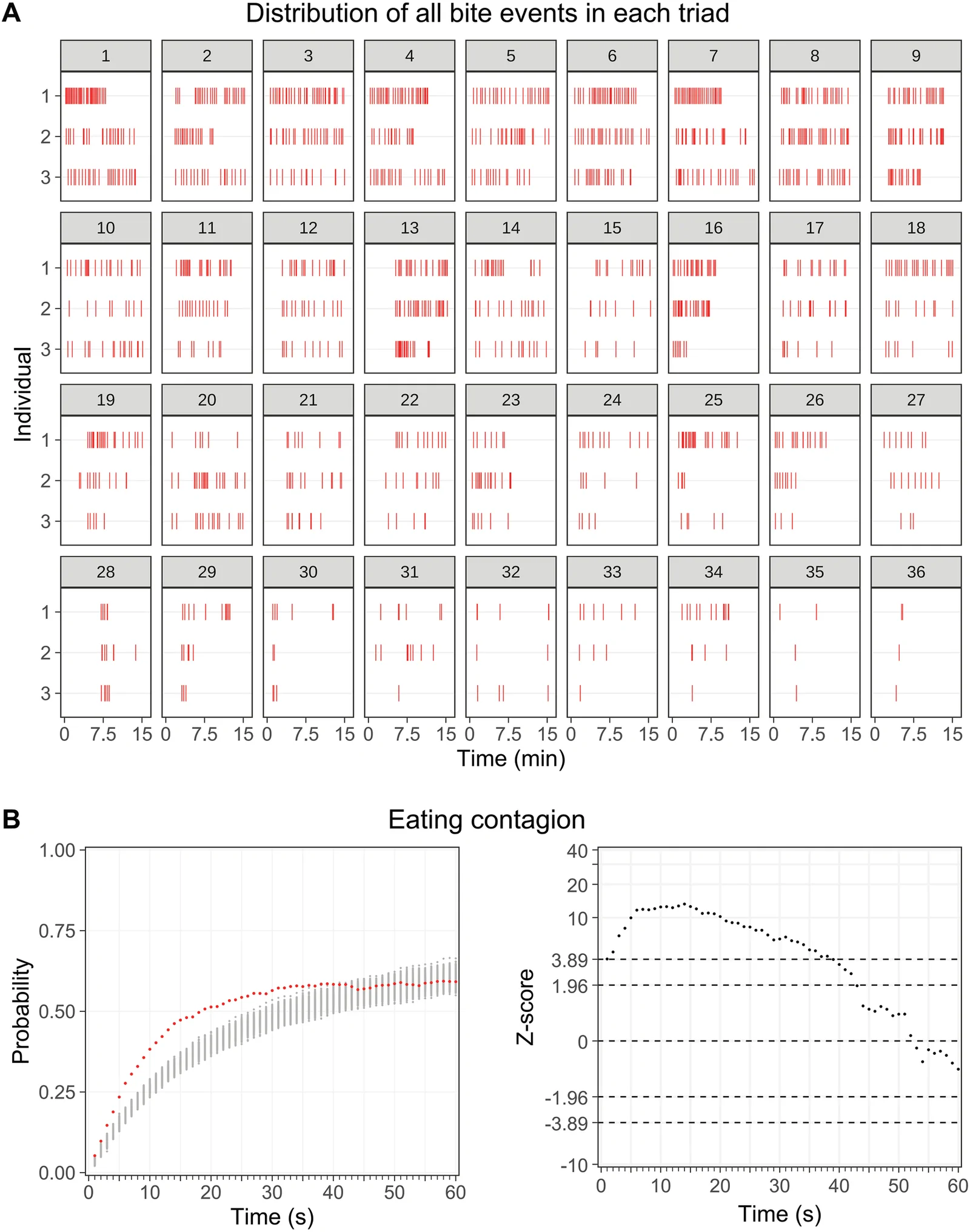
Case 2: Contagion of eating behaviour. **A** Distribution of bite events for each triad (*N* = 1427) across the ~15-minute conversation. Triads (arranged from top left to bottom right) and individuals within each triad (1–3) are ranked by total bite count, and short bite events (≤ 3 s) are slightly expanded to improve visualisation. **B** Left: Observed (red dots) and permuted (grey dots, 1000 permutations) probabilities of eating contagion as a function of latency. **B** Right: Latency-resolved Z-scores quantifying the deviation of observed probabilities from chance. |Z | ≥ 1.96 indicates *p* ≤ 0.05 and |Z | ≥ 3.89 indicates *p* ≤ 0.0001. **B** is based on *N* = 1426 bite events. One event was excluded because it occurred within 1 s of the end of the interaction and could therefore not be evaluated for potential contagion. Statistical inference is based on pointwise, two-sided Z-tests using permutation-derived null distributions generated from 1000 permutations. Pointwise p-values for each cumulative latency threshold are reported in Table S6.

We next examined latency-resolved deviations from chance in eating behaviour under laboratory conditions. The dataset comprised 36 groups (*N*= 108 participants) recorded in person while engaging in 15-minute spontaneous conversations, from which bite event onsets were extracted as discrete event timestamps. The distribution of bite events across conversations is shown in Fig. 5A. In total, 1,427 bite events were recorded, with substantial variation between groups (39.6 ± 23.8 per group, range = 4–82) and across individuals (13.2 ± 9.2 per participant; range = 1–36).

Figure 5B (left panel) shows the observed (red dots) and permuted (grey dots; 1,000 permutations) latency-resolved contagion likelihood across thresholds. The probability that eating by other group members followed a focal bite event increased from 5.26% within the first second to 38.24% within 10 s, 51.35% within 20 s, 56.43% within 30 s, and 58.37% within 40 s. Observed probabilities exceeded those expected under the permutation-based null model across a broad latency range, with elevated values apparent shortly after the focal event and persisting over several tens of seconds.

Statistical inference supported this pattern (Fig. 5B, right panel; Table S6). Significant deviations from chance were detectable within the 0-1 s window (IRR [95% CI] = 1.52 [1.19, 1.98], *Z* = 3.92, *p* < 0.0001), increased up to 14 s (IRR [95% CI] = 1.42 [1.33, 1.51], *Z* = 13.31, *p* < 0.0001), and gradually declined thereafter, remaining significant until 42 s (IRR [95% CI] = 1.07 [1.02, 1.13], *Z* = 2.70, *p* < 0.05). Compared to laughter, eating contagion unfolded over longer timescales, consistent with the slower, more sustained temporal dynamics of eating behaviour.

**Fig. 6:**
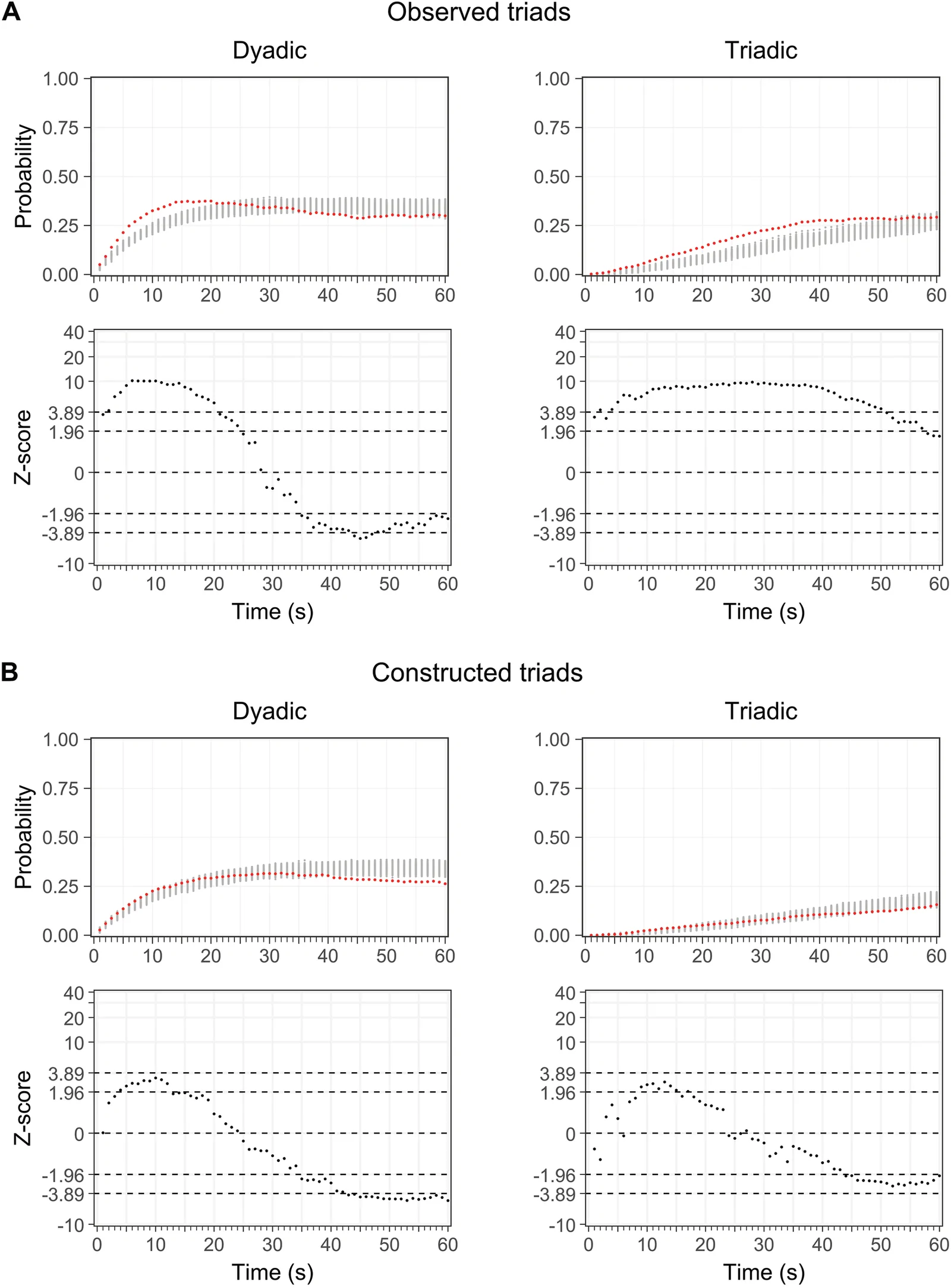
Case 2: Eating contagion in observed and constructed triads. **A** Latency-resolved probabilities of contagion and corresponding Z-scores for observed triads, shown separately for dyadic (left) and triadic (right) effects. **B** Latency-resolved probabilities of contagion and corresponding Z-scores for constructed triads, shown separately for dyadic (left) and triadic (right) effects. In the probability panels (top row), red dots denote observed data and grey dots represent permuted data. Across all panels, ${|Z|}\ge 1.96$ indicates p ≥ 0.05 and |Z|≥ 3.89 indicates *p* ≤ 0.0001. Analyses are based on N = 1426 bite events for observed triads and N = 1411 bite events for constructed triads. Statistical inference is based on pointwise, two-sided Z-tests using permutation-derived null distributions generated from 1000 permutations. Pointwise p-values for each cumulative latency threshold are reported in Tables S7–S10.

Differences between dyadic and triadic effects were examined by differentiating contagion according to the number of responding individuals (Fig. 6A). For dyadic effects (Fig. 6A, left panels; Table S7), significant deviations from the permutation-based null model were detectable within the 0–1 s window: compared with 3.46% expected by chance, biting occurred with a probability of 5.04% (IRR [95% CI] = 1.47 [1.16, 1.95], *Z* = 3.58, *p* < 0.05). The effect increased in magnitude, reaching a maximum at 6 s (24.77% vs. 16.77%; IRR [95% CI] = 1.52 [1.35, 1.63], *Z* = 10.17, *p* < 0.0001), before gradually decreasing and remaining significant until 24 s (36.42% vs. 33.19%; IRR [95% CI] = 1.08 [1.01, 1.16], *Z* = 2.42, *p* < 0.05). For triadic effects (Fig. 6A, right panels; Table S8), significant deviations were also detectable within the first second (0.21% vs. 0.04%; IRR [95% CI] = 5.51 [1.50, >1,000], *Z* = 3.28, *p* < 0.05). Effects peaked at 28 s (8.67% vs. 4.14%; IRR [95% CI] = 1.70 [1.49, 1.99], *Z* = 9.75, *p* < 0.0001) and persisted with reduced magnitude until 57 s (28.57% vs. 24.71%; IRR [95% CI] = 1.12 [1.01, 1.26], *Z* = 3.28, *p* < 0.05). Compared with laughter, dyadic and triadic effects in eating unfolded over longer timescales and were of comparable magnitude across response levels.

Control analyses based on constructed triads, revealed small but statistically significant positive effects both at the dyadic (Table S9) and triadic (Table S10) levels within latency ranges overlapping those observed in the real data. However, these effects were substantially weaker in magnitude and restricted to shorter time windows (4–12 s for dyadic influence and 9–17 s for triadic influence). Both their magnitude and temporal persistence were clearly distinguishable from the more extended effects in the observed triads. By contrast, negative dyadic effects in the real data (Fig. 6A, left panels; Z ≤ -1.96 between 35 and 59 s) were closely mirrored in both magnitude and timing by significant negative effects in the constructed triads (Fig. 6B, left panels). This correspondence suggests that these negative effects are unlikely to reflect inhibitory contagion, but instead may arise from non-social temporal dependencies shared by real and constructed groups.

Taken together, these findings suggest that although short-lived positive deviations can arise under constructed conditions, the more sustained latency-resolved clustering observed in real groups cannot be fully accounted for by chance co-occurrence or baseline event structure alone. To further rule out within-individual temporal dependencies as an alternative explanation, we conducted an additional specificity check assessing within-individual repetition (Supplementary Fig. 1, right column). Eating exhibited reduced within-individual repetition at short latencies, with significantly lower-than-expected probabilities between 1 and 18 s (all IRRs ≤ 0.87, -8.09 ≤ Z ≤ -2.09, *p* ≤ 0.05; Table S12). These findings indicate that short-latency clustering is not attributable to immediate within-individual repetition and support the interpretation that the dyadic and triadic effects reflect inter-individual contagion rather than within-individual temporal dependencies.

## Discussion

We introduced SPREAD, a conceptual and statistical framework designed to address four central methodological challenges in behavioural contagion research. The empirical demonstrations with laughter and eating show how SPREAD distinguishes contagion from coincidental co-occurrence, characterises temporal dynamics without relying on predefined windows, adjusts for behavioural base rates, and accommodates variation in social configuration. By generating latency-resolved contagion profiles that are comparable across behaviours and interaction contexts, the framework extends from dyads to triads and larger groups while preserving group structure.

A central challenge in behavioural contagion research is distinguishing contagion from coincidental temporal overlap. By comparing observed event sequences to permutation-based null expectations, SPREAD provides a systematic framework for quantifying whether temporal clustering between individuals exceeds what would be expected by chance alone. Control analyses using constructed triads, in which systematic social dependencies are eliminated by design, further reduce the likelihood of false-positive detections. For laughter, no sustained effects emerged in the control analysis within the relevant latency range. For eating, an effect was observed which, however, was substantially weaker and temporally more limited than the effect observed in real triads. Overall, these findings support the interpretation that the observed effects were not driven by artefactual temporal structure or baseline event distributions.

In addition to facilitative effects, SPREAD can capture inhibitory contagion dynamics, in which observing a behaviour is associated with a reduced likelihood of subsequent occurrence in others. Although inhibitory patterns have received comparatively less attention in behavioural contagion research, they may represent an important dimension of social influence. For example, recent work has shown that eating can attenuate the contagion of freezing behaviour^26^. By enabling detection of both facilitative and inhibitory deviations from permutation-based expectations, SPREAD allows contagion processes to be examined without presupposing the direction of social influence.

A key implication of the SPREAD framework concerns the temporal structure of behavioural contagion. Fixed-window approaches can mask the temporal structure of contagion and may conflate rapid reflex-like responses with slower, temporally extended processes (e.g., ^15,16^). By estimating contagion strength across a continuous range of temporal lags, SPREAD characterises latency-resolved contagion profiles without imposing predefined latency boundaries. In the present demonstrations, this revealed behaviour-specific temporal signatures: laughter contagion peaked at short latencies but remained detectable over longer intervals, whereas eating contagion unfolded more gradually and persisted across extended timescales. Retaining the full latency-resolved profile facilitates comparison of contagion timing, magnitude, and persistence across behaviours and interaction contexts.

Differences in behavioural base rates represent a central methodological consideration when quantifying behavioural contagion. Because frequently occurring behaviours are more likely to coincide by chance, inference requires null expectations that preserve individual event frequencies while removing cross-individual temporal dependence^7,27^. SPREAD addresses this by comparing contagion estimates with permutation-based null distributions that retain individual base rates while disrupting inter-individual temporal coupling. By basing inference on these permutation-based expectations, the framework reduces susceptibility to frequency-driven artefacts and makes contagion estimates robust to between-individual differences in behavioural frequency.

Group size and social configuration represent an additional dimension of behavioural contagion that is often simplified in dyadic designs. While dyadic approaches have provided important insights, many behaviours unfold in group settings in which multiple individuals may exhibit the target behaviour^28^. In the present triadic demonstrations, contagion effects differed depending on whether responses involved one versus both interaction partners, consistent with the possibility that multi-exposure dynamics may shape transmission likelihood^29^. By preserving group structure, SPREAD enables systematic examination of how contagion varies as a function of the number of responding individuals and can be extended to larger groups or networked interaction contexts. Possible applications range from joint task performance^30^, to collective decision-making^31,32^, and group interactions^26,28,33^.

SPREAD is designed for spontaneous behaviours in which contagion is hypothesised to arise from structured temporal dependencies between behavioural events. Within this scope, the framework is applicable to a range of human and non-human animal behaviours, including laughter^5^, yawning^4,20,27^, facial expressions^8^, vocalisations^34^, and feeding-related behaviours^6,7,15,16,26^. In addition to identifying contagion, the framework provides a basis for distinguishing contagion from social facilitation^2^, a related but conceptually distinct phenomenon. Social facilitation is characterised by elevated behavioural rates at the group level without structured temporal dependencies between individuals, whereas behavioural contagion is reflected in temporal clustering between individuals that exceeds permutation-based expectations.

Like all analytical approaches, SPREAD has limitations that should be considered. The framework assumes that behaviour can be represented as discrete event onsets, which may limit applicability to behaviours with continuous expression or ambiguous temporal boundaries. In addition, the permutation procedure preserves individual event frequencies but treats event times as exchangeable within individuals, and thus does not explicitly model within-individual temporal structure. Where relevant, the null model can be extended by introducing additional permutation constraints to account for refractory periods, habituation, or other sequential dependencies. Future extensions may further broaden applicability by incorporating covariates, modelling time-varying base rates, or adapting the framework to networked interaction settings that integrate social and perceptual context, such as attention measures, speaker-listener roles, relationship structure, or experimentally imposed interaction conditions. Such extensions would enable contagion to be examined not only as an overall temporal dependency but also as a conditional process that varies across interaction partners and contexts.

In sum, SPREAD provides a permutation-based, latency-resolved, and socially structure-sensitive framework for quantifying behavioural contagion in event-based interaction data. By integrating base-rate correction, latency-specific estimation, and social configuration within a unified statistical architecture, the framework supports systematic examination of contagion dynamics across dyadic, triadic, and larger group interactions. More broadly, SPREAD provides a methodological foundation for investigating how behaviours spread through social systems across species and contexts.

## Supplementary information

Transparent Peer Review file

Supplementary Material SPREAD, a framework and open-source R toolbox to quantify behavioural contagion

## Data Availability

The deidentified participant data used in this study is available in the publicly accessible repository KonData under CC BY 4.0 license at https://doi.org/10.48606/bnhwqgyuzsqayz1c^35^.

## Code availability

The analysis code used in this study is available in the publicly accessible repository KonData under CC BY 4.0 license at https://doi.org/10.48606/bnhwqgyuzsqayz1c^35^. SPREAD is implemented in R (version 4.5.2 or higher; https://www.r-project.org) and depends on the following packages: tidyverse, data.table, RcppAlgos, Rfast, patchwork, collapse, and ggpubr. All required packages and installation instructions are listed at the beginning of each script, with full citations provided in the accompanying README file in the KonData Repository^35^.

## References

[1] Lewin, K. Behavior and development as a function of the total situation. in *Manual of child psychology* (ed. Carmichael, L.) 791–844 (John Wiley & Sons, Inc., 1946).

[2] McDougall, P. L. When behaviors are contagious and mimicry matters: a 5-step framework for predicting the spontaneous transmission of automatic behavior. *Comp. cognition Behav. Rev.* **20**, 97–125 (2025).

[3] Parkinson, B. Intragroup emotion convergence: beyond contagion and social appraisal. *Personal. Soc. Psychol. Rev.* **24**, 121–140 (2020). 10.1177/1088868319882596 31642389

[4] Norscia, I. et al. Is it a match? Yawn contagion and smile mimicry in toddlers. *Hum. Nat.* **36**, 70–97 (2025). 10.1007/s12110-025-09488-8 PMC12058824 40080328

[5] Provine, R. R. Contagious laughter: laughter is a sufficient stimulus for laughs and smiles. *Bull. psychonomic Soc.* **30**, 1–4 (1992).

[6] Bell, B. M. et al. Sensing eating mimicry among family members. *Transl. Behav. Med.* **9**, 422–430 (2019). 10.1093/tbm/ibz051 31094447

[7] Gligorić, K. et al. Food choice mimicry on a large university campus. *PNAS nexus* **3**, pgae517 (2024). 10.1093/pnasnexus/pgae517 PMC11645252 39677365

[8] Chartrand, T. L. & Bargh, J. A. The chameleon effect: the perception–behavior link and social interaction. *J. Personal. Soc. Psychol.* **76**, 893–910 (1999). 10.1037//0022-3514.76.6.893 10402679

[9] Chetouani, M., Delaherche, É., Dumas, G. & Cohen, D. Interpersonal synchrony: from social perception to social interaction. in *Social signal processing* (eds Burgoon, J. K., Magnetat-Thalmann, N., Pantic, M. & Vinciarelli, A.) 202–212 (Cambridge University Press, Cambridge, UK, 2017).

[10] daSilva, E. B. & Wood, A. How and why people synchronize: an integrated perspective. *Pers. Soc. Psychol. Rev.* **29**, 159–187 (2025). 10.1177/10888683241252036 38770754

[11] Redcay, E. & Schilbach, L. Using second-person neuroscience to elucidate the mechanisms of social interaction. *Nat. Rev. Neurosci.* **20**, 495–505 (2019). 10.1038/s41583-019-0179-4 PMC6997943 31138910

[12] Schilbach, L. & Redcay, E. Synchrony across brains. *Annu. Rev. Psychol.* **76**, 883–911 (2025). 10.1146/annurev-psych-080123-101149 PMC12668431 39441884

[13] Campbell, M. W. & Cox, C. R. Observational data reveal evidence and parameters of contagious yawning in the behavioral repertoire of captive-reared chimpanzees (*Pan troglodytes*). *Sci. Rep.* **9**, 13271 (2019). 10.1038/s41598-019-49698-6 PMC6744492 31519942

[14] Kapitány, R. & Nielsen, M. Are yawns really contagious? A critique and quantification of yawn contagion. *Adapt. Hum. Behav. Physiol.* **3**, 134–155 (2017).

[15] Hermans, R. C. et al. Mimicry of food intake: the dynamic interplay between eating companions. *PLoS one* **7**, e31027 (2012). 10.1371/journal.pone.0031027 PMC3270030 22312438

[16] Sharps, M. et al. Examining evidence for behavioural mimicry of parental eating by adolescent females. An observational study. *Appetite* **89**, 56–61 (2015). 10.1016/j.appet.2015.01.015 25624021

[17] Onishi, E., Brooks, J., Inoue, S. & Yamamoto, S. Socially contagious urination in chimpanzees. *Curr. Biol.* **35**, R58–R59 (2025). 10.1016/j.cub.2024.11.052 39837269

[18] De Weck, M., Perriard, B., Annoni, J.-M. & Britz, J. Hearing someone laugh and seeing someone yawn: modality-specific contagion of laughter and yawning in the absence of others. *Front. Psychol.* **13**, 780665 (2022). 10.3389/fpsyg.2022.780665 PMC8891493 35250722

[19] Moreland, R. L. Are dyads really groups? *Small group Res.* **41**, 251–267 (2010).

[20] Norscia, I. & Palagi, E. Yawn contagion and empathy in *Homo sapiens*. *PLoS one* **6**, e28472 (2011). 10.1371/journal.pone.0028472 PMC3233580 22163307

[21] R Core Team. R: a language and environment for statistical computing. R Foundation for Statistical Computing (2025).

[22] Halvorson, M. A., McCabe, C. J., Kim, D. S., Cao, X. & King, K. M. Making sense of some odd ratios: a tutorial and improvements to present practices in reporting and visualizing quantities of interest for binary and count outcome models. *Psychol. Addictive Behav.* **36**, 284–295 (2022). 10.1037/adb0000669 PMC8553813 33914563

[23] García-Pérez, M. A. Use and misuse of corrections for multiple testing. *Methods Psychol.* **8**, 100120 (2023).

[24] Phipson, B. & Smyth, G. K. Permutation p-values should never be zero: calculating exact p-values when permutations are randomly drawn. *Stat. Appl. Genet. Mol. Biol.* **9**, 39 (2010). 10.2202/1544-6115.1585 21044043

[25] DiCiccio, T. J. & Efron, B. Bootstrap confidence intervals. *Stat. Sci.* **11**, 189–228 (1996).

[26] Straßheim, J. et al. Food as an Icebreaker: The Impact of Social Eating on Freezing Body movements in Natural Conversations. Preprint at 10.31219/osf.io/tvzcp_v1 (2025).

[27] Valente, D. et al. First evidence of contagious yawning in a wild lemur. *Behav. Ecol. Sociobiol.* **77**, 11 (2022).

[28] Gallup, A. C. et al. Visual attention and the acquisition of information in human crowds. *PNAS* **109**, 7245–7250 (2012). 10.1073/pnas.1116141109 PMC3358867 22529369

[29] Chiba-Okabe, H. & Plotkin, J. B. Social learning with complex contagion. *Proc. Natl. Acad. Sci.* **121**, e2414291121 (2024). 10.1073/pnas.2414291121 PMC11626147 39602255

[30] Meisner, O. C. et al. Diverse and flexible strategies enable successful cooperation in marmoset dyads. *Curr. Biol.* **35**, 4509–4521.e5 (2025). 10.1016/j.cub.2025.08.005 PMC12404675 40882630

[31] Woolley, K. & Fishbach, A. A recipe for friendship: similar food consumption promotes trust and cooperation. *J. Consum. Psychol.* **27**, 1–10 (2017).

[32] Woolley, K. & Fishbach, A. Shared plates, shared minds: consuming from a shared plate promotes cooperation. *Psychol. Sci.* **30**, 541–552 (2019). 10.1177/0956797619830633 30830834

[33] Putra, P. et al. Smile synchrony predicts the quality of children’s peer interactions. Preprint at 10.31234/osf.io/zcpsu_v1 (2026).

[34] Rossetto, F., Cardoso, G. C. & Laiolo, P. Eavesdropping and contagious alarming in bird communities. *Learn Behav.* **53**, 321–334 (2025). 10.3758/s13420-025-00678-z PMC12615574 40457154

[35] Straßheim, J., Bousquet, C. A. H., Köchling, J., Renner, B. & Schupp, H. T. SPREAD - Open-source Toolbox for Quantifying Behavioural Contagion Dynamics. 10.48606/bnhwqgyuzsqayz1c (2026). PMC13650113 42850275

