# Supplementary material for: SPREAD, a framework and open-source R toolbox to quantify behavioural contagion
Source: Commun Psychol. 2026 Oct 8;4:130. doi: 10.1038/s44271-026-00538-0 (PMC13650113; doi:10.1038/s44271-026-00538-0)
Supplement: Supplementary file 1 [file 44271_2026_538_MOESM1_ESM.pdf]

nature portfolio

Peer Review File

SPREAD, a framework and open-source R toolbox to quantify behavioural contagion

Corresponding Author: Dr Jana Straßheim

This file contains all reviewer reports in order by version, followed by all author rebuttals in order by version.

Version 0:

Reviewer comments:

Reviewer #1

(Remarks to the Author)
In this excellent manuscript the authors introduce a methodological approach to quantitatively assessing behavioral contagion.
In my view this is an important contribution that is likely to have a significant impact.
My only criticism is that the authors fail to make reference to relevant work in social neuroscience, even though this field might particularly benefit from the new approach. Schilbach and colleagues (2013, 2019, 2025) have introduced the notion of ‚second-person neuroscience' which focuses heavily on the behavioral dynamics of dyadic social interactions.

Reviewer #2

(Remarks to the Author)
This manuscript presents a nice, robust, useful method to detect behaioural contagion including a description, the r code to implement it and its application to three case studies. I assume this is a Primer submission.

While not conceptually novel in the sense that it is a combination of time series with permutation analyses of behavioural data, this clear implementation of the method and code will be useful for those interested in contagion and other possible applications. By facilitating quantification of contagion, framework has a clear potential to influence thinking in the field. The R code is clearly documented and commented and easy to use, and the examples illustrate the approach well. I recommend accepting this manuscript with a few minor changes, outlined below together.

Comments

32-33 "behavioural contagion, which is defined as the spontaneous spread of behaviours from one person to another". I wonder if this should include a temporal consideration (within a given window of time". Otherwise the definition could be applied at different temporal timescales to language learning; imitation; etc.

38. Add a short review of existing approaches to quantifying contagion, justifying the challenges mentioned in p. 3.

40. [Comment to primary challenge: maybe the situation drives a behaviour (eg late at night, everyone yawns, even without contagion). Is this considered in the model?

45. [Second challenge and linked to 3 challenge: is contagion necessarily close in time? Would we call contagion something like environmental enhancement, whereby being around people who yawn makes me more likely to yawn even if not directly copy any specific person?]

68. A possible extension: Analyse the effect of perception / attention: Could code in the data whether each individual has perceived the action, and then analyse it.

73-76. Could the follower perspective be called "baseline"? That would clarify this concewpt.

125. Grammar "Importantly, social structure, encompassing both group size and relational configuration, represents

analytically distinct dimensions that can be examined independently within the SPREAD framework." Should be "Importantly, both group size and relational configuration aspects of social structure represent analytically distinct dimensions that can be examined independently within the SPREAD framework."

128. How exactly are the extended analyses subbested in lines 125-133 implemented? Can they be done with the current code? or do they need code extension by future users?

134. Figure 2. Mostly clear! But:
- What does "Loop over groups" mean for the input time-series data?
- In the chance probability distribution graphs in Steps 2 and 3 there seems to be a line outside (below) the chance probability between seconds 45 to 55 approx. I imagine it is a chance occurrence, maybe re-run simulation or run more permutations to avoid this confusing line.
- The caption could be more explicit, e.g., "Observed contagion probabilities (in red) are compared with their corresponding null distributions at each latency"
- Permutations: 1,000 permutations is fine to demonstrate the code, but for the publication I would recommend a more robust 10,000 permutations (throughout the paper).

180-181. Typo. Remove "$|Z| < 3.89$ indicates $p < 0.05$" fom captions of Fig. 3, and also from Figs. 4, 5 and 6.

198. "Constructed triads". This term is clearly defined, but I wonder if it would be more intuitive to call these "Control triads" or "Control scrambled triads".

306. The paper could more explicitly mention additional possible applications beyond contagion, e.g. other forms of social learning beyond contagion such as social enhancement, imitation, and overimitation; social media post analysis…

420-443. I can't review the mathematical formulation.

If space is a problem, or for simplicity's sake, I would remove panel A from Figs. 3 and 5. And for the inferential stats, there is no need to show both the probability and the Z-score graphs, since they show the same information, in Figs. 3, 4, 5, and 6. I would remove Probability and leave Z-score graphs only.

Code files: README_SPREAD Open-source Toolbox for Quantifying Behavioural Contagion Dynamics.txt:
Even though the required structure of the input data is mostly clear from the data files, it would be useful to explain exactly what each variable is, e.g. in Bite_Behavioural contagion.csv, what is Event_n?
In the same .txt document, under the description of the files, it could be included which file contains the Statistical inference tests that are mentioned under CODE FUNCTIONALITY AND INSTRUCTIONS FOR USE.

Reviewer #3

(Remarks to the Author)
This article presents a permutation-based time-series framework for quantifying behavioural contagion. By comparing observed temporal co-occurrence against null distributions derived from permuted event sequences it addresses four recurrent methodological challenges: distinguishing genuine contagion from chance, resolving contagion dynamics across latencies rather than arbitrary windows, controlling for base-rate variation, and accommodating social structure at dyadic and group levels.

Overall, the paper is clearly written and methodologically motivated. Its principal contribution lies less in advancing a wholly new mathematical principle than in providing a structured and practical framework for bringing established permutation logic to bear on contagion data in a rigorous and accessible way. This can be very useful to the community but the scope should be clear in the manuscript already in the abstract and introduction. Otherwise the manuscript risks suggesting somewhat overstated novelty. A clearer delineation of which components are drawn from established contagion and event-time methods, which are adapted, and which represent novel contributions specific to SPREAD would strengthen the methodological transparency of the manuscript. Therefore, manuscript should strengthen the literature review around relevant methods, acknowledging prior work and clarify what exactly is new in the mathematical section.

It may also help to clarify the role of the Z-scores. Since the method is permutation-based, readers may wonder whether these are mainly descriptive summaries or the main inferential metric, and why direct permutation p-values were not used. Additionally, claims of broad generality may need to be made with more caution if not supported by clear evidence. Furthermore, the egocentric analysis seems under-integrated conceptually. It would help clarifying the motivation. Finally, it would be useful if the article discuss what other applications could address in real-time social interaction, beyond contagion and how those could be differentiated from contagion per se.

In sum, SPREAD represents a well-implemented and practically valuable contribution to the study of behavioral contagion, one whose impact would be best served by a more measured account of its novelty and a more generous engagement with the methodological landscape it builds upon.

Version 1:

Reviewer comments:

Reviewer #3

(Remarks to the Author)
The authors have satisfactorily addressed my concerns. The revised manuscript is clearer in its framing, methodological contribution, and scope. Therefore, I recommend acceptance.

Warm wishes and best of luck,
Dimitris Bolis

Open Access This Peer Review File is licensed under a Creative Commons Attribution 4.0 International License, which permits use, sharing, adaptation, distribution and reproduction in any medium or format, as long as you give appropriate credit to the original author(s) and the source, provide a link to the Creative Commons license, and indicate if changes were made.
In cases where reviewers are anonymous, credit should be given to 'Anonymous Referee' and the source.
The images or other third party material in this Peer Review File are included in the article's Creative Commons license, unless indicated otherwise in a credit line to the material. If material is not included in the article's Creative Commons license and your intended use is not permitted by statutory regulation or exceeds the permitted use, you will need to obtain permission directly from the copyright holder.
To view a copy of this license, visit https://creativecommons.org/licenses/by/4.0/

Reviewer #1 (Remarks to the Author):

In this excellent manuscript the authors introduce a methodological approach to quantitatively assessing behavioral contagion. In my view this is an important contribution that is likely to have a significant impact.

My only criticism is that the authors fail to make reference to relevant work in social neuroscience, even though this field might particularly benefit from the new approach. Schilbach and colleagues (2013, 2019, 2025) have introduced the notion of ‚second-person neuroscience' which focuses heavily on the behavioral dynamics of dyadic social interactions.

Thank you very much for your positive assessment of our manuscript and for this suggestion. We have addressed it by explicitly incorporating the "second-person neuroscience" framework in the revised Introduction, lines 51-54, citing Redcay and Schilbach (2019) and Schilbach and Redcay (2025). We note that this perspective emphasises behavioural dynamics in real-time social interaction and state that SPREAD can complement this line of work by providing a conceptual and statistical framework, implemented as an open-source R toolbox, for quantifying behavioural contagion in time-series interaction data.

Reviewer #2 (Remarks to the Author):

This manuscript presents a nice, robust, useful method to detect behaioural contagion including a description, the r code to implement it and its application to three case studies. I assume this is a Primer submission.

While not conceptually novel in the sense that it is a combination of time series with permutation analyses of behavioural data, this clear implementation of the method and code will be useful for those interested in contagion and other possible applications. By facilitating quantification of contagion, framework has a clear potential to influence thinking in the field. The R code is clearly documented and commented and easy to use, and the examples illustrate the approach well. I recommend accepting this manuscript with a few minor changes, outlined below together.

We thank Reviewer 2 for the constructive review. We appreciate your positive assessment and have revised the manuscript and documentation accordingly. Below we respond point-by-point.

General framing (Primer/Resource; novelty)

We agree that the main contribution is not conceptual novelty, but a clear analytical framework and open-source implementation. We revised the manuscript to reflect this positioning more clearly and moderated the framing throughout (see e.g. Introduction, lines 56-103).

32-33 (definition; temporal window): *"behavioural contagion, which is defined as the spontaneous spread of behaviours from one person to another". I wonder if this should include a temporal consideration (within a given window of time". Otherwise the definition could be applied at different temporal timescales to language learning; imitation; etc.*

We agree with this suggestion and we revised the definition to include an explicit temporal consideration (milliseconds to minutes) and clarified that SPREAD quantifies latency-resolved effects rather than relying on a predefined or fixed time window, in the Introduction, lines 32-34.

38. (brief review of existing approaches). *Add a short review of existing approaches to quantifying contagion, justifying the challenges mentioned in p. 3.*

As suggested, we expanded the Introduction, lines 56 -103, to briefly summarise common approaches (time-lagged/window-based analyses and permutation-based tests) and used this to motivate the four methodological challenges.

40. (shared context drives behaviour; yawning late at night) *[Comment to primary challenge: maybe the situation drives a behaviour (eg late at night, everyone yawns, even without contagion). Is this considered in the model[2] [3] ?*

Yes - this concern is central to our null model logic. We have now clarified that the permutation-based null model preserves each individual's event frequency (base rate) and interaction duration while disrupting cross-individual temporal coupling (see Method section, lines 127-130 and lines 180-185). We also strengthened control analyses (constructed groups) that remove shared interaction context, helping assess false positives arising from contextual or structural artefacts (see Method section, lines 235-243).

45. (is contagion necessarily close in time; environmental enhancement) *[Second challenge and linked to 3 challenge: is contagion necessarily close in time? Would we call contagion something like environmental enhancement, whereby being around people who yawn makes me more likely to yawn even if not directly copy any specific person?*

We thank the reviewer for this thoughtful question, which touches on an important conceptual distinction. Behavioural contagion, as operationalised in SPREAD, does not require that triggered behaviours occur immediately, but it does require a temporal dependency between specific behavioural events across individuals (see Introduction, lines 32-34 and Method section, lines 126-127, 166-167). Environmental enhancement, by contrast, reflects a general elevation in the probability of a behaviour in a given social context, without a structured temporal dependency between individual behavioural events. This phenomenon is therefore more closely aligned with social facilitation than with behavioural contagion (McDougall, 2025). We clarified this distinction in the revised manuscript, Discussion section lines 625-630, and explicitly note that SPREAD is designed to detect temporally structured dependencies across a latency range rather than broader context-dependent increases in behavioural probability.

McDougall, P. L. (2025). When behaviors are contagious and mimicry matters: A 5-step framework for predicting the spontaneous transmission of automatic behavior.

Comparative Cognition & Behavior Reviews, *20*, 97–125.
https://doi.org/10.3819/CCBR.2025.200001

68. (extension: perception/attention) *A possible extension: Analyse the effect of perception / attention: Could code in the data whether each individual has perceived the action, and then analyse it.*

We agree with the reviewer and have added a paragraph in Limitations/Extensions, lines 638-644, describing how attention/perception data (e.g., gaze, visibility, speaker/listener roles) could be incorporated as permutation constraints or covariates to test contingency on perception.

73-76. (terminology: follower perspective as "baseline") *Could the follower perspective be called "baseline"? That would clarify this concewpt.*

We retained the forward-/backward-looking terminology (leader-follower framing) for clarity, but revised the text to avoid confusion and to explain the "backward-looking" analysis as assessing preceding exposures (see Method section, lines 219-227). We did not adopt the term "baseline" because it could be confused with base rates; instead we clarify the conceptual meaning directly in the Introduction, lines 96-100.

125. Grammar *"Importantly, social structure, encompassing both group size and relational configuration, represents analytically distinct dimensions that can be examined independently within the SPREAD framework." Should be "Importantly, both group size and relational configuration aspects of social structure represent analytically distinct dimensions that can be examined independently within the SPREAD framework."*

Thank you for pointing this out. The sentence and the corresponding paragraph were changed in the process of the revision.

128. (implementation of extended analyses: are they in the current code?) *How exactly are the extended analyses subbested in lines 125-133 implemented? Can they be done with the current code? or do they need code extension by future users?*

We clarified this point in the manuscript (see Method section, lines 219-227) and documentation (README file: Optional Backward Analyses). The extended analyses described in the manuscript can be implemented with the current code, with only minor

adjustments depending on the research question, and these are explained in the code documentation. Analyses of emitter numbers can be conducted directly with the existing implementation. Analyses of social configuration, such as familiar versus unfamiliar individuals, require only a preprocessing step to define the relevant relationships, after which the analysis can be run separately for the corresponding subsets. Forward- and backward-looking analyses are implemented by changing the temporal direction relative to the focal event. To facilitate this, we added an additional example code file demonstrating the backward-looking analysis directly: "Behavioural contagion_Laughter_Backward_1-60s.R".

134. Figure 2. *Mostly clear! But:*

- *What does "Loop over groups" mean for the input time-series data?*

- *In the chance probability distribution graphs in Steps 2 and 3 there seems to be a line outside (below) the chance probability between seconds 45 to 55 approx. I imagine it is a chance occurrence, maybe re-run simulation or run more permutations to avoid this confusing line.*

- *The caption could be more explicit, e.g., "Observed contagion probabilities (in red) are compared with their corresponding null distributions at each latency"*

Thank you for these three helpful suggestions, which substantially improved the clarity and interpretability of Figure 2. In response, we made the following revisions:

- "Loop over groups": we revised the figure legend (lines 149-151) to clarify that this step refers to applying the full analysis procedure independently to each observed group in the input time-series data, with results aggregated across groups at a later stage.
- Outlying line in chance distributions: we revised the illustrative Figure 2 to remove this chance fluctuation, thereby avoiding a potentially confusing visual feature and improving interpretability.
- Caption made more explicit: we revised the caption to introduce the colour convention explicitly (lines 145-147) and to clarify that observed contagion probabilities are compared with their corresponding null distributions at each latency in Step 3 (lines 157-160).

Permutations (1,000 vs. 10,000)*: 1,000 permutations is fine to demonstrate the code, but for the publication I would recommend a more robust 10,000 permutations (throughout the paper).*

We thank the reviewer for this helpful suggestion. During development of the framework, we initially tested the main contagion effects using 10,000 permutations. These analyses yielded highly similar results to those obtained with 1,000 permutations. Because 1,000 permutations provided stable results while substantially reducing computation time, we report the analyses based on 1,000 permutations throughout the manuscript. We now explicitly note in the revised Method section, lines 190-191, that the main effects were evaluated with 10,000 permutations and that the results were highly consistent with those reported using 1,000 permutations. In addition, we now also display the overall contagion for eating and laughter with 1,000 vs 10,000 permutations now in the Supplementary Material, Fig. S2 and S3, so that users can make an informed choice based on their own data and research questions.

180-181. (remove Z-threshold text from captions) *Typo. Remove "$|Z| < 3.89$ indicates $p < 0.05$" fom captions of Fig. 3, and also from Figs. 4, 5 and 6.*

We corrected the typo in the "|Z| …" threshold statements in all figure captions as suggested.

198 (terminology "Constructed triads"). *"Constructed triads". This term is clearly defined, but I wonder if it would be more intuitive to call these "Control triads" or "Control scrambled triads".*

We retained "constructed triads" because it more accurately reflects that these are generated by recombining individuals from the existing dataset (see Method section lines 235-243). We felt "control triads" could imply a separate experimental condition or independently collected comparison group.

306. (additional applications beyond contagion). *The paper could more explicitly mention additional possible applications beyond contagion, e.g. other forms of social learning beyond contagion such as social enhancement, imitation, and overimitation; social media post analysis…*

We revised the Discussion, lines 625-630, to more clearly situate SPREAD in relation to related forms of social influence. We now note that the framework can also be applied to other event-based interaction dynamics (e.g., social enhancement and temporally structured dependencies), while maintaining a clear distinction from the narrower contagion definition used here.

420-443. (mathematical formulation*) I can't review the mathematical formulation.*

We have revised the mathematical formulation to improve clarity and consistency with the Method section and notation, lines 254-332, and ensured that it is fully self-contained.

Figures: removing panels / probability vs Z-score. *If space is a problem, or for simplicity's sake, I would remove panel A from Figs. 3 and 5. And for the inferential stats, there is no need to show both the probability and the Z-score graphs, since they show the same information, in Figs. 3, 4, 5, and 6. I would remove Probability and leave Z-score graphs only.*

Thank you for this helpful suggestion. After careful consideration, we decided to retain panel A in Figures 3 and 5, as well as the probability graphs alongside the Z-score graphs in Figures 3-6. Panel A shows the raw behavioural event distributions and is therefore important for understanding the empirical structure of the data before the permutation-based analysis is applied. Although the probability and Z-score graphs convey related information, they serve complementary purposes: the probability graphs illustrate the underlying observed and permuted distributions directly, whereas the Z-score graphs provide the corresponding standardised inferential summary. We therefore believe that showing both improves the didactic value and interpretability of the figures, particularly for readers seeking to understand how the statistical inference follows from the permutation procedure.

Code files: Code/README documentation. *README_SPREAD Open-source Toolbox for Quantifying Behavioural Contagion Dynamics.txt: Even though the required structure of the input data is mostly clear from the data files, it would be useful to explain exactly what each variable is, e.g. in Bite_Behavioural contagion.csv, what is Event_n? In the same .txt document, under the description of the files, it could be included which file contains the Statistical inference tests that are mentioned under CODE FUNCTIONALITY AND INSTRUCTIONS FOR USE.*

We appreciate this helpful suggestion and have revised the README file accordingly. Specifically, we now provide a clearer description of the required input data structure and define each variable, including Event_n in the example data files (see "Codebook"). We have also amended the file description section to indicate which script contains the statistical inference tests referred to under "Code functionality and instructions for use."

Reviewer #3 (Remarks to the Author):

This article presents a permutation-based time-series framework for quantifying behavioural contagion. By comparing observed temporal co-occurrence against null distributions derived from permuted event sequences it addresses four recurrent methodological challenges: distinguishing genuine contagion from chance, resolving contagion dynamics across latencies rather than arbitrary windows, controlling for base-rate variation, and accommodating social structure at dyadic and group levels.

1) *Overall, the paper is clearly written and methodologically motivated. Its principal contribution lies less in advancing a wholly new mathematical principle than in providing a structured and practical framework for bringing established permutation logic to bear on contagion data in a rigorous and accessible way.*

Thank you for your careful and constructive review. We particularly appreciate your balanced assessment of the manuscript's contribution and your concrete guidance on scope, novelty, and methodological transparency. We followed these recommendations closely in the revision and revised the framing to reflect this positioning (see Introduction, lines 55-106).

2) *This can be very useful to the community but the scope should be clear in the manuscript already in the abstract and introduction. Otherwise the manuscript risks suggesting somewhat overstated novelty.*

We agree and revised the Abstract (lines 15-20; 22-26) and Introduction (lines 55-65; 101-110) to provide a more measured account of novelty and scope. We now clearly state that SPREAD integrates established time-series and permutation logic into a unified, implementable framework addressing four recurring challenges, and we moderated broad generality claims unless directly supported by the demonstrations.

3) *A clearer delineation of which components are drawn from established contagion and event-time methods, which are adapted, and which represent novel contributions specific to SPREAD would strengthen the methodological transparency of the manuscript.*

We addressed this by explicitly delineating (i) components that reflect established event-time and permutation logic, (ii) components adapted for latency-resolved contagion profiling, and (iii) contributions specific to SPREAD as an integrated framework, see Introduction, lines 55-116, that combines latency-resolved estimation, base-rate correction, decomposition of contagion by number of responding group members, and analyses sensitive to social configuration and directionality (forward- and backward-looking perspectives) within a single open-source R toolbox.

4) *Therefore, manuscript should strengthen the literature review around relevant methods, acknowledging prior work and clarify what exactly is new in the mathematical section.*

We expanded the Introduction to more explicitly acknowledge relevant prior approaches (time-lagged analyses and earlier permutation-based applications and used this review to motivate the four recurring challenges that SPREAD integrates (see Introduction, lines 55-65; lines 101-103). We revised the framing around the mathematical formulation to make clear what is standard (event-time representation; permutation-based null expectations, see Method section, lines 254-332), what is adapted (latency-resolved estimation across a continuous range of thresholds, see Method section, lines 126-130), and what is specific to SPREAD (the integration of the four challenges within one analytical pipeline, including the differentiation of dyadic and higher-order contagion effects within groups, the assessment of forward- and backward-directional effects, and configuration-sensitive analyses, see Method section, lines 208-213).

5) *It may also help to clarify the role of the Z-scores. Since the method is permutation-based, readers may wonder whether these are mainly descriptive summaries or the main inferential metric, and why direct permutation p-values were not used.*

We clarified that Z-scores in SPREAD serve as the primary standardised inferential summary (computed relative to the permutation-derived null distribution at each latency, see Method section, lines 193-204; lines 300-302). We report Z-scores (and IRR as a

complementary effect-size measure, see lines 303-320) because they provide a continuous, interpretable metric for comparing effect magnitude across behaviours, datasets, and social configurations, while retaining permutation-based inference.

6) *Additionally, claims of broad generality may need to be made with more caution if not supported by clear evidence*

We agree and moderated statements of generality throughout the Abstract (lines 22-26), Introduction (lines 108-116) and Discussion (lines 621-627; 632-634; 645-651), restricting claims to what is supported by the framework design and the presented demonstrations.

7) *Furthermore, the egocentric analysis seems under-integrated conceptually. It would help clarifying the motivation.*

We agree with this point and revised the manuscript to motivate the within-individual analysis explicitly as a specificity check distinguishing inter-individual contagion from within-individual temporal clustering (see Method section, lines 228-234; lines 244-253). We also integrated this check directly within the case-study validation narrative, see Case 1, lines 478-484 and Case 2, lines 547-554, rather than treating it as a separate add-on.

8) *Finally, it would be useful if the article discuss what other applications could address in real-time social interaction, beyond contagion and how those could be differentiated from contagion per se.*

We expanded the Discussion, lines 622-627 and lines 636-644, to note additional applications to other event-based interaction dynamics and clarified how these can be differentiated from contagion as defined here (temporally structured inter-individual dependence exceeding permutation-based expectations) versus context-driven rate changes without structured temporal coupling.
