# Supplementary material for: SPREAD, a framework and open-source R toolbox to quantify behavioural contagion
Source: Commun Psychol. 2026 Oct 8;4:130. doi: 10.1038/s44271-026-00538-0 (PMC13650113; doi:10.1038/s44271-026-00538-0)
Supplement: Supplementary file 2 [file 44271_2026_538_MOESM2_ESM.pdf]

Supplementary Material

SPREAD, a framework and open-source R toolbox to quantify behavioural contagion

Jana Straßheim[1,2*], Christophe Bousquet[1,2*], Johanna Köchling[1,2],

Britta Renner[1,2], Harald T. Schupp[1,2]

[1] Department of Psychology, University of Konstanz, Universitätsstraße 10, 78464 Konstanz, Germany

[2] Centre for the Advanced Study of Collective Behaviour, University of Konstanz, Universitätsstraße 10, 78464 Konstanz, Germany

[*]These authors contributed equally: Jana Straßheim, Christophe Bousquet

Corresponding author e-mail: jana.strassheim@uni-konstanz.de

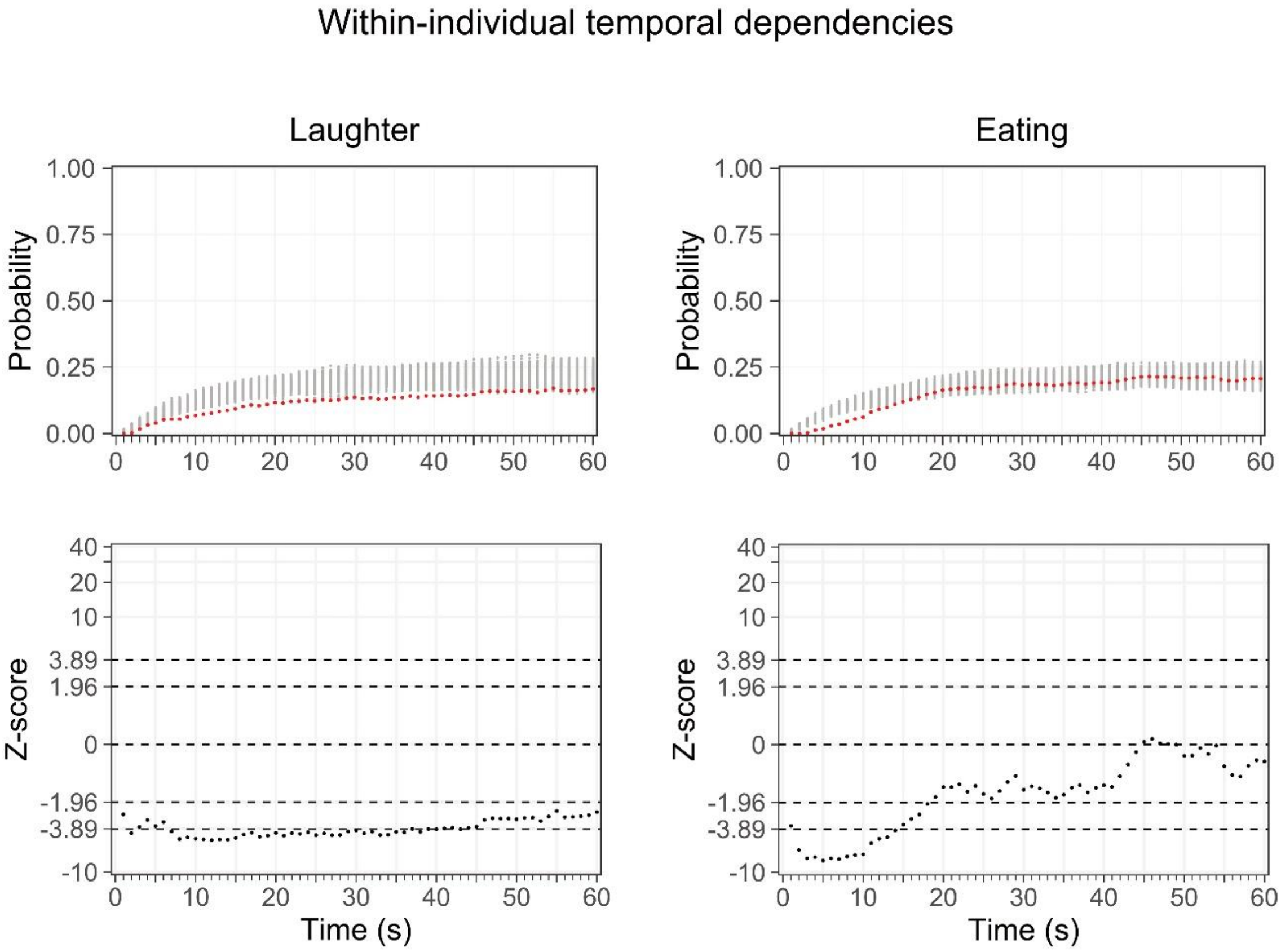

Supplementary Figure 1. **Within-individual temporal dependencies in laughter and eating.** Left and right panels show latency-resolved probabilities and corresponding Z-scores for within-individual self-repetition of laughter and bite events, respectively. In the probability panels (top row), red dots represent observed data and grey dots represent permuted data. In the Z-score panels (bottom row), $|Z| \geq 1.96$ indicates $p \leq .05$, and $|Z| \geq 3.89$ indicates $p \leq .0001$. Analyses are based on N = 1,031 laughter events and N = 1,426 bite events. Statistical inference is based on pointwise, two-sided Z-tests using permutation-derived null distributions generated from 1,000 permutations. Pointwise p-values for each cumulative latency threshold are reported in Tables S11-S12.

Table S1. Statistics for the overall contagion of laughter in the observed triads.

Latency	Influence type	N total	N observed	N expected	SD expected	Z-score	p	IRR	IRR 95%CI low	IRR 95%CI high
1	Overall	1031	176	36.05	5.67	24.70	<0.0001	4.88	3.67	7.04
2	Overall	1026	254	70.55	7.34	24.99	<0.0001	3.60	3.02	4.47
3	Overall	1007	294	100.16	8.37	23.16	<0.0001	2.94	2.50	3.48
4	Overall	984	316	125.63	9.27	20.53	<0.0001	2.52	2.20	2.94
5	Overall	963	322	147.80	10.01	17.40	<0.0001	2.18	1.93	2.51
6	Overall	948	328	168.03	10.25	15.61	<0.0001	1.95	1.74	2.21
7	Overall	926	335	184.31	10.19	14.79	<0.0001	1.82	1.64	2.03
8	Overall	905	328	198.38	10.16	12.76	<0.0001	1.65	1.50	1.82
9	Overall	889	334	211.75	10.28	11.89	<0.0001	1.58	1.44	1.74
10	Overall	868	332	222.13	10.29	10.68	<0.0001	1.49	1.37	1.65
11	Overall	850	342	231.27	10.29	10.77	<0.0001	1.48	1.36	1.63
12	Overall	830	337	238.35	10.26	9.61	<0.0001	1.41	1.31	1.54
13	Overall	808	333	243.64	10.30	8.67	<0.0001	1.37	1.26	1.48
14	Overall	784	329	246.99	10.28	7.98	<0.0001	1.33	1.23	1.44
15	Overall	772	326	252.87	10.08	7.26	<0.0001	1.29	1.19	1.40
16	Overall	766	333	260.08	10.00	7.29	<0.0001	1.28	1.19	1.38
17	Overall	760	338	266.60	10.09	7.08	<0.0001	1.27	1.18	1.37

18	Overall	743	331	268.43	10.01	6.25	<0.0001	1.23	1.15	1.33
19	Overall	730	332	271.33	9.93	6.11	<0.0001	1.22	1.14	1.32
20	Overall	721	329	274.97	9.89	5.46	<0.0001	1.20	1.12	1.29
21	Overall	707	324	276.13	9.82	4.87	<0.0001	1.17	1.09	1.26
22	Overall	696	318	277.86	9.67	4.15	<0.0001	1.14	1.07	1.23
23	Overall	683	316	278.29	9.50	3.97	<0.0001	1.14	1.06	1.21
24	Overall	669	317	277.84	9.29	4.21	<0.0001	1.14	1.07	1.22
25	Overall	653	309	276.20	9.26	3.54	<0.001	1.12	1.05	1.20
26	Overall	639	306	275.03	9.10	3.40	<0.001	1.11	1.04	1.19
27	Overall	631	303	275.91	9.11	2.97	0.003	1.10	1.03	1.17
28	Overall	622	301	276.07	9.17	2.72	0.007	1.09	1.02	1.17
29	Overall	608	296	273.75	9.00	2.47	0.013	1.08	1.01	1.15
30	Overall	596	295	271.93	9.01	2.56	0.010	1.08	1.02	1.16
31	Overall	585	293	270.63	8.69	2.57	0.010	1.08	1.02	1.16
32	Overall	572	286	267.84	8.58	2.11	0.034	1.07	1.00	1.14
33	Overall	563	287	266.65	8.49	2.40	0.017	1.08	1.01	1.15
34	Overall	552	279	264.35	8.55	1.71	0.087	1.06	0.99	1.13
35	Overall	539	268	260.80	8.55	0.84	0.400	1.03	0.97	1.10
36	Overall	533	264	260.44	8.45	0.42	0.673	1.01	0.95	1.08
37	Overall	524	262	258.49	8.40	0.42	0.676	1.01	0.95	1.08

38	Overall	513	256	255.47	8.43	0.06	0.949	1.00	0.94	1.07
39	Overall	508	256	255.17	8.48	0.10	0.922	1.00	0.94	1.07
40	Overall	501	255	254.00	8.52	0.12	0.907	1.00	0.94	1.07
41	Overall	496	251	253.52	8.52	-0.30	0.768	0.99	0.93	1.07
42	Overall	485	248	249.99	8.44	-0.24	0.813	0.99	0.93	1.07
43	Overall	479	245	248.74	8.38	-0.45	0.655	0.98	0.93	1.06
44	Overall	472	242	246.90	8.37	-0.59	0.558	0.98	0.92	1.06
45	Overall	470	245	247.59	8.43	-0.31	0.758	0.99	0.93	1.06
46	Overall	458	236	243.05	8.15	-0.87	0.387	0.97	0.91	1.04
47	Overall	445	228	237.70	7.97	-1.22	0.223	0.96	0.90	1.03
48	Overall	435	225	233.77	7.78	-1.13	0.260	0.96	0.91	1.03
49	Overall	433	225	234.13	7.82	-1.17	0.243	0.96	0.90	1.03
50	Overall	424	222	230.59	7.67	-1.12	0.263	0.96	0.91	1.03
51	Overall	418	218	228.63	7.74	-1.37	0.170	0.95	0.90	1.02
52	Overall	411	216	225.91	7.62	-1.30	0.193	0.96	0.90	1.03
53	Overall	406	215	224.31	7.63	-1.22	0.222	0.96	0.90	1.03
54	Overall	401	212	222.68	7.63	-1.40	0.162	0.95	0.89	1.02
55	Overall	399	211	222.63	7.63	-1.52	0.127	0.95	0.89	1.02
56	Overall	391	211	219.29	7.53	-1.10	0.271	0.96	0.90	1.04
57	Overall	384	209	216.40	7.42	-1.00	0.318	0.97	0.90	1.04

58	Overall	377	205	213.37	7.43	-1.13	0.260	0.96	0.90	1.03
59	Overall	373	203	212.10	7.40	-1.23	0.219	0.96	0.90	1.03
60	Overall	369	200	210.79	7.44	-1.45	0.147	0.95	0.89	1.02

Note. Latency refers to the number of seconds after a focal laughter was emitted, Influence type refers to the type of contagion under consideration, N total refers to the number of laughter events relevant for this specific latency, N observed refers to the observed number of cases in which at least one other individual laughed after the focal laughter within the given latency, N expected refers to the average number of expected cases over 1,000 permutations of the observed time series, SD expected is the associated standard deviation, Z-score refers to the Z-score computed as (N observed - N expected) / SD expected, p is the associated p-value, IRR refers to the Incidence Rate Ratio, a measure of effect size computed as N observed / N expected, IRR 95%CI low and IRR 95%CI high refer to the lower and the higher end of the associated 95% Confidence Interval respectively. Statistical inference is based on pointwise, two-sided Z-tests using latency-specific null distributions generated from 1,000 permutations.

Table S2. Statistics for the contagion of laughter at the dyadic level in the observed triads.

Latency	Influence type	N total	N observed	N expected	SD expected	Z-score	p	IRR	IRR 95%CI low	IRR 95%CI high
1	Dyadic	1031	153	35.69	5.58	21.01	<0.0001	4.29	3.26	6.12
2	Dyadic	1026	216	69.00	7.17	20.50	<0.0001	3.13	2.61	3.93
3	Dyadic	1007	249	96.97	8.16	18.62	<0.0001	2.57	2.20	3.06
4	Dyadic	984	264	120.27	9.01	15.96	<0.0001	2.20	1.91	2.58
5	Dyadic	963	261	139.88	9.58	12.65	<0.0001	1.87	1.64	2.15
6	Dyadic	948	263	157.18	9.72	10.89	<0.0001	1.67	1.48	1.89
7	Dyadic	926	262	170.46	9.52	9.62	<0.0001	1.54	1.39	1.74
8	Dyadic	905	255	181.37	9.40	7.84	<0.0001	1.41	1.28	1.56
9	Dyadic	889	255	191.53	9.54	6.65	<0.0001	1.33	1.21	1.48
10	Dyadic	868	251	198.84	9.47	5.51	<0.0001	1.26	1.15	1.39
11	Dyadic	850	261	204.76	9.39	5.99	<0.0001	1.27	1.17	1.40
12	Dyadic	830	252	208.90	9.35	4.61	<0.0001	1.21	1.11	1.31
13	Dyadic	808	244	211.16	9.33	3.52	<0.001	1.16	1.07	1.26
14	Dyadic	784	241	211.87	9.40	3.10	0.002	1.14	1.05	1.24
15	Dyadic	772	235	214.59	9.17	2.22	0.026	1.10	1.01	1.20
16	Dyadic	766	240	218.39	9.21	2.35	0.019	1.10	1.01	1.19
17	Dyadic	760	240	221.55	9.21	2.00	0.045	1.08	1.00	1.18

18	Dyadic	743	234	220.86	9.18	1.43	0.152	1.06	0.98	1.15
19	Dyadic	730	238	221.08	9.04	1.87	0.061	1.08	1.00	1.17
20	Dyadic	721	231	221.71	9.00	1.03	0.302	1.04	0.97	1.13
21	Dyadic	707	228	220.53	8.96	0.83	0.405	1.03	0.95	1.12
22	Dyadic	696	217	219.73	8.88	-0.31	0.758	0.99	0.91	1.07
23	Dyadic	683	217	217.91	8.72	-0.10	0.917	1.00	0.92	1.08
24	Dyadic	669	219	215.39	8.57	0.42	0.674	1.02	0.94	1.10
25	Dyadic	653	212	212.16	8.53	-0.02	0.985	1.00	0.93	1.08
26	Dyadic	639	210	209.30	8.49	0.08	0.934	1.00	0.93	1.09
27	Dyadic	631	203	208.14	8.55	-0.60	0.548	0.98	0.90	1.06
28	Dyadic	622	206	206.38	8.71	-0.04	0.965	1.00	0.92	1.09
29	Dyadic	608	203	202.82	8.57	0.02	0.983	1.00	0.93	1.09
30	Dyadic	596	201	199.60	8.61	0.16	0.871	1.01	0.93	1.10
31	Dyadic	585	197	196.70	8.05	0.04	0.970	1.00	0.93	1.09
32	Dyadic	572	191	192.97	7.84	-0.25	0.802	0.99	0.92	1.08
33	Dyadic	563	190	190.42	7.71	-0.05	0.956	1.00	0.92	1.09
34	Dyadic	552	180	187.23	7.71	-0.94	0.348	0.96	0.89	1.04
35	Dyadic	539	172	183.34	7.76	-1.46	0.144	0.94	0.87	1.02
36	Dyadic	533	170	181.61	7.74	-1.50	0.133	0.94	0.86	1.02
37	Dyadic	524	169	178.80	7.62	-1.29	0.198	0.95	0.87	1.03

38	Dyadic	513	164	175.40	7.59	-1.50	0.133	0.93	0.86	1.02
39	Dyadic	508	163	173.85	7.59	-1.43	0.153	0.94	0.86	1.02
40	Dyadic	501	162	171.75	7.69	-1.27	0.205	0.94	0.87	1.03
41	Dyadic	496	156	170.15	7.79	-1.82	0.069	0.92	0.84	1.00
42	Dyadic	485	154	166.53	7.60	-1.65	0.099	0.92	0.85	1.01
43	Dyadic	479	152	164.50	7.56	-1.65	0.099	0.92	0.84	1.01
44	Dyadic	472	149	162.16	7.47	-1.76	0.078	0.92	0.84	1.00
45	Dyadic	470	149	161.45	7.47	-1.67	0.095	0.92	0.84	1.01
46	Dyadic	458	143	157.34	7.13	-2.01	0.044	0.91	0.83	1.00
47	Dyadic	445	140	152.70	7.01	-1.81	0.070	0.92	0.84	1.01
48	Dyadic	435	140	149.20	6.77	-1.36	0.174	0.94	0.86	1.03
49	Dyadic	433	139	148.49	6.69	-1.42	0.156	0.94	0.85	1.02
50	Dyadic	424	136	145.23	6.64	-1.39	0.165	0.94	0.85	1.02
51	Dyadic	418	133	142.96	6.63	-1.50	0.133	0.93	0.85	1.02
52	Dyadic	411	132	140.32	6.46	-1.29	0.198	0.94	0.86	1.03
53	Dyadic	406	129	138.44	6.44	-1.47	0.143	0.93	0.84	1.02
54	Dyadic	401	123	136.60	6.43	-2.12	0.034	0.90	0.82	0.99
55	Dyadic	399	123	135.78	6.45	-1.98	0.048	0.91	0.82	0.99
56	Dyadic	391	122	132.81	6.39	-1.69	0.090	0.92	0.83	1.01
57	Dyadic	384	122	130.22	6.31	-1.30	0.193	0.94	0.85	1.03

58	Dyadic	377	119	127.65	6.39	-1.35	0.175	0.93	0.85	1.03
59	Dyadic	373	117	126.16	6.38	-1.44	0.151	0.93	0.84	1.03
60	Dyadic	369	117	124.65	6.39	-1.20	0.231	0.94	0.85	1.04

Note. Latency refers to the number of seconds after a focal laughter was emitted, Influence type refers to the type of contagion under consideration, N total refers to the number of laughter events relevant for this specific latency, N observed refers to the observed number of cases in which at least one other individual laughed after the focal laughter within the given latency, N expected refers to the average number of expected cases over 1,000 permutations of the observed time series, SD expected is the associated standard deviation, Z-score refers to the Z-score computed as (N observed - N expected) / SD expected, p is the associated p-value, IRR refers to the Incidence Rate Ratio, a measure of effect size computed as N observed / N expected, IRR 95%CI low and IRR 95%CI high refer to the lower and the higher end of the associated 95% Confidence Interval respectively. Statistical inference is based on pointwise, two-sided Z-tests using latency-specific null distributions generated from 1,000 permutations.

Table S3. Statistics for the contagion of laughter at the triadic level in the observed triads.

Latency	Influence type	N total	N observed	N expected	SD expected	Z-score	p	IRR	IRR 95%CI low	IRR 95%CI high
1	Triadic	1031	23	0.37	0.62	36.50	<0.0001	62.68	11.50	>1,000
2	Triadic	1026	38	1.54	1.24	29.36	<0.0001	24.65	9.51	>1,000
3	Triadic	1007	45	3.19	1.76	23.81	<0.0001	14.10	6.54	>1,000
4	Triadic	984	52	5.36	2.22	21.02	<0.0001	9.71	5.36	27.08
5	Triadic	963	61	7.92	2.59	20.52	<0.0001	7.70	4.55	21.26
6	Triadic	948	65	10.85	3.02	17.93	<0.0001	5.99	3.81	11.50
7	Triadic	926	73	13.85	3.41	17.34	<0.0001	5.27	3.53	9.81
8	Triadic	905	73	17.01	3.71	15.10	<0.0001	4.29	3.01	7.22
9	Triadic	889	79	20.22	4.06	14.47	<0.0001	3.91	2.79	6.20
10	Triadic	868	81	23.29	4.36	13.23	<0.0001	3.48	2.54	5.36
11	Triadic	850	81	26.51	4.59	11.86	<0.0001	3.06	2.29	4.57
12	Triadic	830	85	29.45	4.73	11.75	<0.0001	2.89	2.20	4.14
13	Triadic	808	89	32.47	4.80	11.79	<0.0001	2.74	2.12	3.79
14	Triadic	784	88	35.12	4.99	10.59	<0.0001	2.51	1.93	3.50
15	Triadic	772	91	38.28	5.22	10.10	<0.0001	2.38	1.84	3.20
16	Triadic	766	93	41.68	5.42	9.46	<0.0001	2.23	1.77	2.94
17	Triadic	760	98	45.05	5.62	9.42	<0.0001	2.18	1.74	2.89

18	Triadic	743	97	47.57	5.75	8.60	<0.0001	2.04	1.65	2.67
19	Triadic	730	94	50.25	5.82	7.51	<0.0001	1.87	1.52	2.42
20	Triadic	721	98	53.26	5.90	7.59	<0.0001	1.84	1.51	2.35
21	Triadic	707	96	55.60	5.98	6.75	<0.0001	1.73	1.42	2.16
22	Triadic	696	101	58.12	6.07	7.06	<0.0001	1.74	1.42	2.15
23	Triadic	683	99	60.39	6.07	6.36	<0.0001	1.64	1.35	2.01
24	Triadic	669	98	62.45	6.02	5.90	<0.0001	1.57	1.31	1.90
25	Triadic	653	97	64.05	6.07	5.43	<0.0001	1.51	1.27	1.84
26	Triadic	639	96	65.74	6.04	5.01	<0.0001	1.46	1.24	1.74
27	Triadic	631	100	67.77	6.03	5.34	<0.0001	1.48	1.25	1.77
28	Triadic	622	95	69.69	6.26	4.04	<0.0001	1.36	1.14	1.64
29	Triadic	608	93	70.93	6.20	3.56	<0.001	1.31	1.10	1.58
30	Triadic	596	94	72.33	6.04	3.59	<0.001	1.30	1.11	1.56
31	Triadic	585	96	73.93	5.85	3.77	<0.001	1.30	1.12	1.53
32	Triadic	572	95	74.87	5.99	3.36	<0.001	1.27	1.09	1.50
33	Triadic	563	97	76.23	6.12	3.40	<0.001	1.27	1.10	1.52
34	Triadic	552	99	77.13	6.15	3.55	<0.001	1.28	1.11	1.53
35	Triadic	539	96	77.46	6.14	3.02	0.003	1.24	1.07	1.48
36	Triadic	533	94	78.83	6.23	2.44	0.015	1.19	1.03	1.42
37	Triadic	524	93	79.69	6.22	2.14	0.032	1.17	1.01	1.39

38	Triadic	513	92	80.06	6.08	1.96	0.050	1.15	1.00	1.35
39	Triadic	508	93	81.32	6.06	1.93	0.054	1.14	0.99	1.34
40	Triadic	501	93	82.26	6.22	1.73	0.084	1.13	0.98	1.33
41	Triadic	496	95	83.37	6.29	1.85	0.064	1.14	0.99	1.33
42	Triadic	485	94	83.47	6.22	1.69	0.091	1.13	0.98	1.33
43	Triadic	479	93	84.25	6.34	1.38	0.167	1.10	0.96	1.29
44	Triadic	472	93	84.75	6.25	1.32	0.187	1.10	0.96	1.28
45	Triadic	470	96	86.14	6.39	1.54	0.123	1.11	0.97	1.31
46	Triadic	458	93	85.71	6.38	1.14	0.253	1.09	0.95	1.27
47	Triadic	445	88	85.00	6.31	0.48	0.635	1.04	0.90	1.22
48	Triadic	435	85	84.58	6.29	0.07	0.946	1.00	0.88	1.18
49	Triadic	433	86	85.64	6.30	0.06	0.955	1.00	0.88	1.17
50	Triadic	424	86	85.36	6.25	0.10	0.918	1.01	0.88	1.17
51	Triadic	418	85	85.67	6.26	-0.11	0.915	0.99	0.87	1.16
52	Triadic	411	84	85.59	6.26	-0.25	0.800	0.98	0.86	1.14
53	Triadic	406	86	85.87	6.28	0.02	0.984	1.00	0.88	1.17
54	Triadic	401	89	86.07	6.21	0.47	0.638	1.03	0.91	1.21
55	Triadic	399	88	86.85	6.27	0.18	0.855	1.01	0.89	1.18
56	Triadic	391	89	86.48	6.30	0.40	0.689	1.03	0.90	1.20
57	Triadic	384	87	86.18	6.28	0.13	0.896	1.01	0.88	1.19

58	Triadic	377	86	85.72	6.31	0.04	0.965	1.00	0.88	1.18
59	Triadic	373	86	85.93	6.34	0.01	0.992	1.00	0.87	1.17
60	Triadic	369	83	86.14	6.29	-0.50	0.618	0.96	0.84	1.12

Note. Latency refers to the number of seconds after a focal laughter was emitted, Influence type refers to the type of contagion under consideration, N total refers to the number of laughter events relevant for this specific latency, N observed refers to the observed number of cases in which at least one other individual laughed after the focal laughter within the given latency, N expected refers to the average number of expected cases over 1,000 permutations of the observed time series, SD expected is the associated standard deviation, Z-score refers to the Z-score computed as (N observed - N expected) / SD expected, p is the associated p-value, IRR refers to the Incidence Rate Ratio, a measure of effect size computed as N observed / N expected, IRR 95%CI low and IRR 95%CI high refer to the lower and the higher end of the associated 95% Confidence Interval respectively. Statistical inference is based on pointwise, two-sided Z-tests using latency-specific null distributions generated from 1,000 permutations.

Table S4. Statistics for the contagion of laughter at the dyadic level in the constructed triads.

Latency	Influence type	N total	N observed	N expected	SD expected	Z-score	p	IRR	IRR 95%CI low	IRR 95%CI high
1	Dyadic	989	33	24.20	4.64	1.90	0.058	1.36	0.97	2.06
2	Dyadic	988	50	47.72	6.23	0.37	0.715	1.05	0.83	1.39
3	Dyadic	985	66	68.96	7.09	-0.42	0.676	0.96	0.79	1.20
4	Dyadic	977	80	87.64	7.65	-1.00	0.318	0.91	0.78	1.10
5	Dyadic	973	103	105.13	8.04	-0.26	0.791	0.98	0.85	1.14
6	Dyadic	968	118	120.83	8.31	-0.34	0.734	0.98	0.86	1.13
7	Dyadic	960	125	134.36	8.66	-1.08	0.280	0.93	0.82	1.06
8	Dyadic	956	140	147.19	9.2	-0.78	0.435	0.95	0.84	1.08
9	Dyadic	946	154	157.96	9.38	-0.42	0.673	0.97	0.87	1.10
10	Dyadic	936	169	167.30	9.45	0.18	0.857	1.01	0.91	1.14
11	Dyadic	922	177	175.06	9.64	0.20	0.841	1.01	0.91	1.13
12	Dyadic	916	187	183.03	9.61	0.41	0.680	1.02	0.93	1.14
13	Dyadic	898	192	187.82	9.39	0.44	0.657	1.02	0.93	1.13
14	Dyadic	880	195	191.38	9.49	0.38	0.703	1.02	0.93	1.12
15	Dyadic	873	201	196.73	9.49	0.45	0.653	1.02	0.93	1.12
16	Dyadic	861	206	200.43	9.48	0.59	0.557	1.03	0.94	1.13
17	Dyadic	854	209	204.48	9.43	0.48	0.632	1.02	0.94	1.12

18	Dyadic	844	208	207.43	9.53	0.06	0.952	1.00	0.92	1.10
19	Dyadic	829	204	208.54	9.53	-0.48	0.634	0.98	0.90	1.07
20	Dyadic	814	205	209.20	9.34	-0.45	0.653	0.98	0.90	1.07
21	Dyadic	803	204	210.26	9.50	-0.66	0.510	0.97	0.89	1.06
22	Dyadic	789	203	210.34	9.28	-0.79	0.429	0.97	0.89	1.05
23	Dyadic	779	202	210.95	9.42	-0.95	0.342	0.96	0.88	1.05
24	Dyadic	765	196	210.12	9.30	-1.52	0.129	0.93	0.86	1.02
25	Dyadic	752	193	209.35	9.29	-1.76	0.078	0.92	0.85	1.01
26	Dyadic	742	190	209.07	9.27	-2.06	0.040	0.91	0.84	1.00
27	Dyadic	731	194	208.34	9.15	-1.57	0.117	0.93	0.85	1.02
28	Dyadic	720	196	207.54	9.16	-1.26	0.208	0.94	0.87	1.03
29	Dyadic	707	198	205.63	9.07	-0.84	0.400	0.96	0.88	1.05
30	Dyadic	698	199	204.84	9.10	-0.64	0.521	0.97	0.89	1.06
31	Dyadic	692	195	205.27	9.20	-1.12	0.264	0.95	0.87	1.04
32	Dyadic	679	191	202.85	9.17	-1.29	0.196	0.94	0.86	1.03
33	Dyadic	671	196	201.70	9.02	-0.63	0.528	0.97	0.89	1.06
34	Dyadic	663	193	200.43	9.07	-0.82	0.412	0.96	0.88	1.05
35	Dyadic	647	188	196.74	8.92	-0.98	0.327	0.96	0.88	1.04
36	Dyadic	640	190	195.42	8.91	-0.61	0.543	0.97	0.89	1.06
37	Dyadic	627	185	192.24	8.8	-0.82	0.410	0.96	0.88	1.05

38	Dyadic	619	184	190.54	8.68	-0.75	0.451	0.97	0.88	1.06
39	Dyadic	609	180	188.28	8.62	-0.96	0.337	0.96	0.88	1.04
40	Dyadic	599	175	185.85	8.48	-1.28	0.201	0.94	0.86	1.03
41	Dyadic	588	171	183.06	8.54	-1.41	0.158	0.93	0.85	1.02
42	Dyadic	578	168	180.45	8.52	-1.46	0.144	0.93	0.85	1.02
43	Dyadic	573	166	179.39	8.48	-1.58	0.114	0.93	0.85	1.03
44	Dyadic	570	164	178.78	8.55	-1.73	0.084	0.92	0.84	1.02
45	Dyadic	568	164	178.51	8.62	-1.68	0.092	0.92	0.84	1.02
46	Dyadic	557	160	175.38	8.41	-1.83	0.067	0.91	0.84	1.01
47	Dyadic	542	151	170.83	8.26	-2.4	0.016	0.88	0.81	0.98
48	Dyadic	532	147	167.91	8.17	-2.56	0.011	0.88	0.80	0.97
49	Dyadic	526	147	166.26	8.11	-2.38	0.018	0.88	0.81	0.98
50	Dyadic	520	145	164.48	8.15	-2.39	0.017	0.88	0.81	0.97
51	Dyadic	513	142	162.47	7.98	-2.57	0.010	0.87	0.80	0.96
52	Dyadic	508	141	161.00	7.91	-2.53	0.011	0.88	0.80	0.97
53	Dyadic	507	140	160.81	7.93	-2.62	0.009	0.87	0.79	0.96
54	Dyadic	501	142	158.92	7.92	-2.14	0.033	0.89	0.81	0.99
55	Dyadic	491	137	155.79	7.71	-2.44	0.015	0.88	0.80	0.98
56	Dyadic	483	135	153.25	7.69	-2.37	0.018	0.88	0.80	0.98
57	Dyadic	478	134	151.67	7.66	-2.31	0.021	0.88	0.80	0.98

58	Dyadic	470	130	149.10	7.61	-2.51	0.012	0.87	0.79	0.96
59	Dyadic	464	131	147.04	7.62	-2.1	0.035	0.89	0.81	0.99
60	Dyadic	461	128	146.03	7.66	-2.35	0.019	0.88	0.79	0.97

Note. Latency refers to the number of seconds after a focal laughter was emitted, Influence type refers to the type of contagion under consideration, N total refers to the number of laughter events relevant for this specific latency, N observed refers to the observed number of cases in which at least one other individual laughed after the focal laughter within the given latency, N expected refers to the average number of expected cases over 1,000 permutations of the observed time series, SD expected is the associated standard deviation, Z-score refers to the Z-score computed as (N observed - N expected) / SD expected, p is the associated p-value, IRR refers to the Incidence Rate Ratio, a measure of effect size computed as N observed / N expected, IRR 95%CI low and IRR 95%CI high refer to the lower and the higher end of the associated 95% Confidence Interval respectively. Statistical inference is based on pointwise, two-sided Z tests using latency-specific null distributions generated from 1,000 permutations.

Table S5. Statistics for the contagion of laughter at the triadic level in the constructed triads.

Latency	Influence type	N total	N observed	N expected	SD expected	Z-score	p	IRR	IRR 95%CI low	IRR 95%CI high
1	Triadic	989	0	0.13	0.36	-0.36	0.717	0.00	0.00	0.00
2	Triadic	988	0	0.49	0.70	-0.70	0.481	0.00	0.00	0.00
3	Triadic	985	1	1.02	1.01	-0.02	0.983	0.98	0.33	>1,000
4	Triadic	977	2	1.77	1.29	0.18	0.860	1.13	0.40	>1,000
5	Triadic	973	4	2.58	1.59	0.89	0.374	1.55	0.67	>1,000
6	Triadic	968	4	3.58	1.83	0.23	0.819	1.12	0.50	4.03
7	Triadic	960	5	4.70	2.11	0.14	0.886	1.06	0.55	4.99
8	Triadic	956	7	5.97	2.44	0.42	0.673	1.17	0.63	3.49
9	Triadic	946	7	7.30	2.64	-0.11	0.909	0.96	0.54	2.35
10	Triadic	936	8	8.70	2.86	-0.25	0.806	0.92	0.56	2.01
11	Triadic	922	8	10.04	3.07	-0.66	0.508	0.80	0.50	1.62
12	Triadic	916	10	11.59	3.22	-0.49	0.621	0.86	0.55	1.67
13	Triadic	898	10	13.01	3.34	-0.90	0.367	0.77	0.50	1.45
14	Triadic	880	12	14.38	3.45	-0.69	0.490	0.83	0.56	1.53
15	Triadic	873	16	15.87	3.60	0.04	0.971	1.01	0.69	1.78
16	Triadic	861	17	17.28	3.75	-0.08	0.940	0.98	0.69	1.72
17	Triadic	854	18	18.81	3.89	-0.21	0.836	0.96	0.67	1.54

18	Triadic	844	18	20.27	4.02	-0.56	0.573	0.89	0.63	1.40
19	Triadic	829	18	21.66	4.06	-0.90	0.367	0.83	0.59	1.23
20	Triadic	814	18	22.98	4.14	-1.20	0.229	0.78	0.57	1.15
21	Triadic	803	20	24.39	4.13	-1.06	0.288	0.82	0.61	1.18
22	Triadic	789	19	25.66	4.27	-1.56	0.119	0.74	0.55	1.08
23	Triadic	779	21	26.98	4.30	-1.39	0.165	0.78	0.58	1.11
24	Triadic	765	24	28.20	4.41	-0.95	0.341	0.85	0.65	1.23
25	Triadic	752	27	29.29	4.54	-0.51	0.613	0.92	0.71	1.34
26	Triadic	742	28	30.55	4.60	-0.55	0.580	0.92	0.71	1.31
27	Triadic	731	29	31.71	4.63	-0.59	0.558	0.91	0.71	1.28
28	Triadic	720	29	32.77	4.66	-0.81	0.419	0.88	0.69	1.22
29	Triadic	707	30	33.80	4.78	-0.80	0.426	0.89	0.69	1.23
30	Triadic	698	31	34.98	4.83	-0.82	0.410	0.89	0.70	1.22
31	Triadic	692	34	36.56	5.01	-0.51	0.609	0.93	0.72	1.24
32	Triadic	679	33	37.46	5.00	-0.89	0.372	0.88	0.70	1.17
33	Triadic	671	35	38.66	5.06	-0.72	0.469	0.91	0.72	1.21
34	Triadic	663	35	39.72	5.12	-0.92	0.357	0.88	0.71	1.17
35	Triadic	647	36	40.27	5.13	-0.83	0.405	0.89	0.71	1.17
36	Triadic	640	37	41.35	5.15	-0.84	0.399	0.89	0.71	1.17
37	Triadic	627	39	41.93	5.15	-0.57	0.569	0.93	0.75	1.21

38	Triadic	619	40	42.80	5.17	-0.54	0.588	0.93	0.76	1.22
39	Triadic	609	39	43.46	5.21	-0.86	0.392	0.90	0.73	1.18
40	Triadic	599	41	44.08	5.21	-0.59	0.554	0.93	0.76	1.21
41	Triadic	588	41	44.56	5.24	-0.68	0.497	0.92	0.75	1.20
42	Triadic	578	40	45.13	5.30	-0.97	0.333	0.89	0.73	1.14
43	Triadic	573	40	46.00	5.39	-1.11	0.266	0.87	0.71	1.12
44	Triadic	570	41	47.05	5.43	-1.11	0.265	0.87	0.71	1.13
45	Triadic	568	41	48.13	5.53	-1.29	0.197	0.85	0.70	1.09
46	Triadic	557	41	48.43	5.55	-1.34	0.181	0.85	0.69	1.08
47	Triadic	542	42	48.38	5.43	-1.17	0.241	0.87	0.72	1.10
48	Triadic	532	41	48.69	5.43	-1.42	0.157	0.84	0.70	1.07
49	Triadic	526	42	49.27	5.37	-1.35	0.176	0.85	0.71	1.08
50	Triadic	520	42	49.80	5.39	-1.45	0.148	0.84	0.70	1.07
51	Triadic	513	42	50.17	5.34	-1.53	0.126	0.84	0.70	1.05
52	Triadic	508	42	50.81	5.40	-1.63	0.103	0.83	0.69	1.04
53	Triadic	507	43	51.76	5.46	-1.61	0.108	0.83	0.69	1.04
54	Triadic	501	41	52.20	5.43	-2.06	0.039	0.79	0.65	0.98
55	Triadic	491	40	52.22	5.41	-2.26	0.024	0.77	0.63	0.96
56	Triadic	483	40	52.37	5.44	-2.27	0.023	0.76	0.64	0.96
57	Triadic	478	41	52.78	5.44	-2.17	0.030	0.78	0.65	0.98

58	Triadic	470	42	52.88	5.44	-2.00	0.045	0.79	0.66	1.00
59	Triadic	464	41	53.19	5.41	-2.25	0.024	0.77	0.64	0.96
60	Triadic	461	41	53.80	5.45	-2.35	0.019	0.76	0.63	0.94

Note. Latency refers to the number of seconds after a focal laughter was emitted, Influence type refers to the type of contagion under consideration, N total refers to the number of laughter events relevant for this specific latency, N observed refers to the observed number of cases in which at least one other individual laughed after the focal laughter within the given latency, N expected refers to the average number of expected cases over 1,000 permutations of the observed time series, SD expected is the associated standard deviation, Z-score refers to the Z-score computed as (N observed - N expected) / SD expected, p is the associated p-value, IRR refers to the Incidence Rate Ratio, a measure of effect size computed as N observed / N expected, IRR 95%CI low and IRR 95%CI high refer to the lower and the higher end of the associated 95% Confidence Interval respectively. Statistical inference is based on pointwise, two-sided Z-tests using latency-specific null distributions generated from 1,000 permutations.

Table S6. Statistics for the overall contagion of eating in the observed triads.

Latency	Influence type	N total	N observed	N expected	SD expected	Z-score	p	IRR	IRR 95%CI low	IRR 95%CI high
1	Overall	1426	75	49.46	6.52	3.92	<0.0001	1.52	1.19	1.98
2	Overall	1424	139	97.26	9.01	4.63	<0.0001	1.43	1.21	1.74
3	Overall	1418	208	140.90	9.99	6.72	<0.0001	1.48	1.30	1.72
4	Overall	1413	266	180.54	10.76	7.94	<0.0001	1.47	1.32	1.67
5	Overall	1408	330	216.99	11.36	9.95	<0.0001	1.52	1.38	1.70
6	Overall	1401	388	250.27	11.76	11.71	<0.0001	1.55	1.42	1.70
7	Overall	1388	424	279.86	12.10	11.91	<0.0001	1.52	1.40	1.65
8	Overall	1376	453	306.95	12.38	11.80	<0.0001	1.48	1.37	1.60
9	Overall	1360	485	330.69	12.78	12.08	<0.0001	1.47	1.36	1.59
10	Overall	1344	514	351.65	12.97	12.52	<0.0001	1.46	1.36	1.57
11	Overall	1319	531	368.33	12.91	12.60	<0.0001	1.44	1.35	1.55
12	Overall	1299	548	384.37	13.27	12.33	<0.0001	1.43	1.34	1.53
13	Overall	1281	567	398.88	13.15	12.79	<0.0001	1.42	1.33	1.52
14	Overall	1249	577	407.24	12.75	13.31	<0.0001	1.42	1.33	1.51
15	Overall	1216	575	413.32	12.80	12.63	<0.0001	1.39	1.31	1.48
16	Overall	1178	566	415.87	12.48	12.03	<0.0001	1.36	1.28	1.44
17	Overall	1156	558	422.19	12.43	10.93	<0.0001	1.32	1.25	1.40

18	Overall	1130	561	425.80	12.27	11.02	<0.0001	1.32	1.25	1.40
19	Overall	1106	561	429.32	12.14	10.84	<0.0001	1.31	1.24	1.38
20	Overall	1073	551	427.74	12.07	10.21	<0.0001	1.29	1.22	1.36
21	Overall	1047	539	427.99	11.94	9.30	<0.0001	1.26	1.19	1.33
22	Overall	1021	534	427.06	11.93	8.97	<0.0001	1.25	1.18	1.32
23	Overall	994	530	424.75	11.87	8.86	<0.0001	1.25	1.18	1.32
24	Overall	969	519	422.61	11.73	8.22	<0.0001	1.23	1.16	1.30
25	Overall	937	509	416.50	11.34	8.16	<0.0001	1.22	1.16	1.29
26	Overall	903	494	408.51	11.25	7.60	<0.0001	1.21	1.15	1.28
27	Overall	882	490	405.80	11.08	7.60	<0.0001	1.21	1.15	1.28
28	Overall	866	481	405.11	11.09	6.84	<0.0001	1.19	1.13	1.26
29	Overall	846	469	401.76	10.97	6.13	<0.0001	1.17	1.11	1.24
30	Overall	824	465	396.81	10.90	6.25	<0.0001	1.17	1.11	1.24
31	Overall	804	460	391.58	10.47	6.53	<0.0001	1.17	1.12	1.24
32	Overall	793	454	391.12	10.45	6.02	<0.0001	1.16	1.10	1.22
33	Overall	773	446	385.87	10.29	5.85	<0.0001	1.16	1.10	1.22
34	Overall	748	433	377.63	10.08	5.50	<0.0001	1.15	1.09	1.21
35	Overall	730	421	372.72	9.91	4.87	<0.0001	1.13	1.07	1.19
36	Overall	720	418	371.49	9.76	4.76	<0.0001	1.13	1.07	1.18
37	Overall	702	406	365.88	9.65	4.16	<0.0001	1.11	1.05	1.17

38	Overall	681	398	358.50	9.43	4.19	<0.0001	1.11	1.06	1.17
39	Overall	668	391	354.82	9.34	3.87	<0.001	1.10	1.05	1.16
40	Overall	651	380	348.91	9.09	3.42	<0.001	1.09	1.04	1.15
41	Overall	628	366	339.39	8.88	3.00	0.003	1.08	1.03	1.14
42	Overall	607	354	330.81	8.60	2.70	0.007	1.07	1.02	1.13
43	Overall	588	339	322.87	8.38	1.92	0.054	1.05	1.00	1.11
44	Overall	573	325	316.98	8.22	0.97	0.330	1.03	0.98	1.08
45	Overall	567	323	315.95	8.27	0.85	0.394	1.02	0.97	1.08
46	Overall	559	320	313.53	8.30	0.78	0.436	1.02	0.97	1.08
47	Overall	553	320	312.19	8.20	0.95	0.341	1.03	0.98	1.08
48	Overall	544	316	309.08	8.13	0.85	0.395	1.02	0.97	1.08
49	Overall	535	311	305.90	8.06	0.63	0.527	1.02	0.97	1.07
50	Overall	526	308	302.57	7.95	0.68	0.495	1.02	0.97	1.07
51	Overall	516	304	298.60	7.89	0.68	0.493	1.02	0.97	1.07
52	Overall	504	294	293.15	7.76	0.11	0.913	1.00	0.95	1.06
53	Overall	486	283	284.13	7.61	-0.15	0.882	1.00	0.94	1.05
54	Overall	483	280	283.79	7.62	-0.50	0.618	0.99	0.93	1.04
55	Overall	475	279	280.43	7.61	-0.19	0.851	0.99	0.94	1.05
56	Overall	467	275	277.07	7.58	-0.27	0.785	0.99	0.94	1.05
57	Overall	459	272	273.66	7.51	-0.22	0.825	0.99	0.94	1.05

58	Overall	455	270	272.44	7.55	-0.32	0.746	0.99	0.94	1.04
59	Overall	449	266	270.02	7.48	-0.54	0.591	0.99	0.93	1.04
60	Overall	441	261	266.39	7.36	-0.73	0.464	0.98	0.93	1.04

Note. Latency refers to the number of seconds after a focal bite was emitted, Influence type refers to the type of contagion under consideration, N total refers to the number of bite events relevant for this specific latency, N observed refers to the observed number of cases in which at least one other individual took a bite after the focal bite event within the given latency, N expected refers to the average number of expected cases over 1,000 permutations of the observed time series, SD expected is the associated standard deviation, Z-score refers to the Z-score computed as (N observed - N expected) / SD expected, p is the associated p-value, IRR refers to the Incidence Rate Ratio, a measure of effect size computed as N observed / N expected, IRR 95%CI low and IRR 95%CI high refer to the lower and the higher end of the associated 95% Confidence Interval respectively. Statistical inference is based on pointwise, two-sided Z-tests using latency-specific null distributions generated from 1,000 permutations.

Table S7. Statistics for the contagion of eating at the dyadic level in the observed triads.

Latency	Influence type	N total	N observed	N expected	SD expected	Z-score	p	IRR	IRR 95%CI low	IRR 95%CI high
1	Dyadic	1426	72	48.92	6.44	3.58	<0.001	1.47	1.16	1.95
2	Dyadic	1424	131	95.25	8.73	4.09	<0.0001	1.38	1.16	1.65
3	Dyadic	1418	197	136.53	9.55	6.33	<0.0001	1.44	1.27	1.68
4	Dyadic	1413	247	172.96	10.27	7.21	<0.0001	1.43	1.28	1.60
5	Dyadic	1408	301	205.63	10.80	8.83	<0.0001	1.46	1.33	1.63
6	Dyadic	1401	347	234.44	11.07	10.17	<0.0001	1.48	1.35	1.63
7	Dyadic	1388	374	259.19	11.38	10.08	<0.0001	1.44	1.33	1.58
8	Dyadic	1376	398	281.24	11.60	10.07	<0.0001	1.42	1.31	1.54
9	Dyadic	1360	420	299.70	11.96	10.05	<0.0001	1.40	1.30	1.52
10	Dyadic	1344	437	315.08	12.09	10.09	<0.0001	1.39	1.29	1.51
11	Dyadic	1319	441	326.33	11.90	9.64	<0.0001	1.35	1.26	1.46
12	Dyadic	1299	449	336.83	12.31	9.11	<0.0001	1.33	1.25	1.43
13	Dyadic	1281	456	345.68	12.14	9.09	<0.0001	1.32	1.23	1.42
14	Dyadic	1249	461	349.02	11.86	9.44	<0.0001	1.32	1.24	1.41
15	Dyadic	1216	451	350.28	11.95	8.43	<0.0001	1.29	1.21	1.38
16	Dyadic	1178	440	348.59	11.55	7.91	<0.0001	1.26	1.19	1.35
17	Dyadic	1156	427	349.94	11.58	6.65	<0.0001	1.22	1.15	1.31

18	Dyadic	1130	421	349.12	11.44	6.28	<0.0001	1.21	1.13	1.29
19	Dyadic	1106	414	348.21	11.33	5.81	<0.0001	1.19	1.12	1.27
20	Dyadic	1073	402	343.11	11.27	5.22	<0.0001	1.17	1.10	1.25
21	Dyadic	1047	380	339.56	11.16	3.62	<0.001	1.12	1.05	1.20
22	Dyadic	1021	371	335.02	11.03	3.26	0.001	1.11	1.04	1.18
23	Dyadic	994	362	329.51	11.11	2.92	0.003	1.10	1.03	1.17
24	Dyadic	969	351	324.56	10.93	2.42	0.016	1.08	1.01	1.16
25	Dyadic	937	335	316.36	10.70	1.74	0.081	1.06	0.99	1.13
26	Dyadic	903	319	307.00	10.39	1.15	0.248	1.04	0.98	1.11
27	Dyadic	882	314	301.84	10.16	1.20	0.232	1.04	0.98	1.11
28	Dyadic	866	299	298.29	10.09	0.07	0.944	1.00	0.94	1.07
29	Dyadic	846	288	292.84	9.94	-0.49	0.626	0.98	0.92	1.05
30	Dyadic	824	281	286.15	9.81	-0.53	0.599	0.98	0.92	1.05
31	Dyadic	804	278	279.92	9.41	-0.20	0.838	0.99	0.93	1.06
32	Dyadic	793	269	276.56	9.26	-0.82	0.414	0.97	0.91	1.04
33	Dyadic	773	263	270.04	9.09	-0.77	0.439	0.97	0.92	1.04
34	Dyadic	748	251	261.57	8.92	-1.19	0.236	0.96	0.90	1.02
35	Dyadic	730	237	255.70	8.80	-2.13	0.034	0.93	0.87	0.99
36	Dyadic	720	232	252.46	8.86	-2.31	0.021	0.92	0.86	0.99
37	Dyadic	702	218	246.29	8.72	-3.25	0.001	0.89	0.83	0.95

38	Dyadic	681	215	238.99	8.49	-2.83	0.005	0.90	0.84	0.97
39	Dyadic	668	209	234.39	8.47	-3.00	0.003	0.89	0.83	0.96
40	Dyadic	651	200	228.31	8.23	-3.44	<0.001	0.88	0.82	0.94
41	Dyadic	628	193	219.96	7.90	-3.41	<0.001	0.88	0.82	0.94
42	Dyadic	607	186	212.37	7.60	-3.47	<0.001	0.88	0.82	0.94
43	Dyadic	588	178	205.28	7.52	-3.63	<0.001	0.87	0.80	0.93
44	Dyadic	573	168	199.80	7.35	-4.33	<0.0001	0.84	0.78	0.90
45	Dyadic	567	163	197.50	7.34	-4.70	<0.0001	0.83	0.77	0.89
46	Dyadic	559	161	194.34	7.31	-4.56	<0.0001	0.83	0.77	0.89
47	Dyadic	553	162	191.86	7.31	-4.08	<0.0001	0.84	0.78	0.91
48	Dyadic	544	161	188.38	7.23	-3.79	<0.001	0.85	0.79	0.92
49	Dyadic	535	158	184.90	7.16	-3.75	<0.001	0.85	0.79	0.92
50	Dyadic	526	157	181.43	7.15	-3.41	<0.001	0.87	0.80	0.94
51	Dyadic	516	157	177.67	7.20	-2.87	0.004	0.88	0.81	0.95
52	Dyadic	504	152	172.92	7.03	-2.98	0.003	0.88	0.81	0.95
53	Dyadic	486	147	166.26	6.97	-2.76	0.006	0.88	0.81	0.96
54	Dyadic	483	142	164.79	6.89	-3.31	<0.001	0.86	0.79	0.93
55	Dyadic	475	142	161.63	6.88	-2.85	0.004	0.88	0.81	0.95
56	Dyadic	467	138	158.44	6.87	-2.98	0.003	0.87	0.79	0.95
57	Dyadic	459	139	155.28	6.84	-2.38	0.017	0.90	0.82	0.97

58	Dyadic	455	139	153.43	6.78	-2.13	0.033	0.91	0.83	0.99
59	Dyadic	449	136	150.94	6.73	-2.22	0.026	0.90	0.82	0.99
60	Dyadic	441	132	147.91	6.69	-2.38	0.017	0.89	0.82	0.98

Note. Latency refers to the number of seconds after a focal bite was emitted, Influence type refers to the type of contagion under consideration, N total refers to the number of bite events relevant for this specific latency, N observed refers to the observed number of cases in which at least one other individual took a bite after the focal bite event within the given latency, N expected refers to the average number of expected cases over 1,000 permutations of the observed time series, SD expected is the associated standard deviation, Z-score refers to the Z-score computed as (N observed - N expected) / SD expected, p is the associated p-value, IRR refers to the Incidence Rate Ratio, a measure of effect size computed as N observed / N expected, IRR 95%CI low and IRR 95%CI high refer to the lower and the higher end of the associated 95% Confidence Interval respectively. Statistical inference is based on pointwise, two-sided Z-tests using latency-specific null distributions generated from 1,000 permutations.

Table S8. Statistics for the contagion of eating at the triadic level in the observed triads.

Latency	Influence type	N total	N observed	N expected	SD expected	Z-score	p	IRR	IRR 95%CI low	IRR 95%CI high
1	Triadic	1426	3	0.54	0.75	3.28	0.001	5.51	1.50	>1,000
2	Triadic	1424	8	2.02	1.43	4.18	<0.0001	3.97	1.60	>1,000
3	Triadic	1418	11	4.37	2.11	3.14	0.002	2.51	1.22	11.00
4	Triadic	1413	19	7.58	2.70	4.24	<0.0001	2.51	1.45	6.33
5	Triadic	1408	29	11.35	3.30	5.36	<0.0001	2.55	1.60	5.74
6	Triadic	1401	41	15.83	3.79	6.64	<0.0001	2.59	1.69	4.51
7	Triadic	1388	50	20.67	4.48	6.54	<0.0001	2.42	1.66	4.06
8	Triadic	1376	55	25.71	4.96	5.90	<0.0001	2.14	1.53	3.38
9	Triadic	1360	65	31.00	5.36	6.34	<0.0001	2.10	1.57	3.08
10	Triadic	1344	77	36.57	5.69	7.10	<0.0001	2.11	1.60	2.94
11	Triadic	1319	90	42.00	6.05	7.94	<0.0001	2.14	1.67	2.98
12	Triadic	1299	99	47.55	6.40	8.04	<0.0001	2.08	1.65	2.83
13	Triadic	1281	111	53.20	6.88	8.40	<0.0001	2.09	1.68	2.84
14	Triadic	1249	116	58.22	7.10	8.14	<0.0001	1.99	1.62	2.62
15	Triadic	1216	124	63.04	7.08	8.61	<0.0001	1.97	1.62	2.52
16	Triadic	1178	126	67.27	6.94	8.46	<0.0001	1.87	1.55	2.33
17	Triadic	1156	131	72.25	7.23	8.12	<0.0001	1.81	1.50	2.26

18	Triadic	1130	140	76.68	7.40	8.56	<0.0001	1.83	1.53	2.22
19	Triadic	1106	147	81.11	7.68	8.58	<0.0001	1.81	1.53	2.21
20	Triadic	1073	149	84.62	7.69	8.37	<0.0001	1.76	1.49	2.12
21	Triadic	1047	159	88.43	7.76	9.10	<0.0001	1.80	1.52	2.15
22	Triadic	1021	163	92.05	7.84	9.05	<0.0001	1.77	1.51	2.12
23	Triadic	994	168	95.24	7.84	9.29	<0.0001	1.76	1.51	2.10
24	Triadic	969	168	98.05	7.80	8.97	<0.0001	1.71	1.47	2.02
25	Triadic	937	174	100.14	7.79	9.48	<0.0001	1.74	1.50	2.05
26	Triadic	903	175	101.51	7.69	9.55	<0.0001	1.72	1.49	2.02
27	Triadic	882	176	103.96	7.65	9.42	<0.0001	1.69	1.47	1.99
28	Triadic	866	182	106.82	7.71	9.75	<0.0001	1.70	1.49	1.99
29	Triadic	846	181	108.92	7.80	9.24	<0.0001	1.66	1.46	1.94
30	Triadic	824	184	110.66	7.70	9.53	<0.0001	1.66	1.47	1.93
31	Triadic	804	182	111.66	7.60	9.25	<0.0001	1.63	1.45	1.89
32	Triadic	793	185	114.55	7.65	9.21	<0.0001	1.61	1.43	1.86
33	Triadic	773	183	115.82	7.54	8.91	<0.0001	1.58	1.41	1.81
34	Triadic	748	182	116.06	7.31	9.02	<0.0001	1.57	1.40	1.78
35	Triadic	730	184	117.02	7.50	8.93	<0.0001	1.57	1.39	1.79
36	Triadic	720	186	119.03	7.62	8.79	<0.0001	1.56	1.39	1.78
37	Triadic	702	188	119.59	7.57	9.03	<0.0001	1.57	1.39	1.80

38	Triadic	681	183	119.50	7.36	8.63	<0.0001	1.53	1.36	1.74
39	Triadic	668	182	120.43	7.24	8.50	<0.0001	1.51	1.34	1.71
40	Triadic	651	180	120.60	7.29	8.15	<0.0001	1.49	1.33	1.69
41	Triadic	628	173	119.43	7.11	7.53	<0.0001	1.45	1.29	1.64
42	Triadic	607	168	118.44	6.99	7.09	<0.0001	1.42	1.27	1.61
43	Triadic	588	161	117.59	6.93	6.27	<0.0001	1.37	1.23	1.54
44	Triadic	573	157	117.18	6.88	5.79	<0.0001	1.34	1.19	1.51
45	Triadic	567	160	118.45	6.99	5.94	<0.0001	1.35	1.21	1.52
46	Triadic	559	159	119.19	7.00	5.69	<0.0001	1.33	1.19	1.50
47	Triadic	553	158	120.33	7.00	5.38	<0.0001	1.31	1.18	1.47
48	Triadic	544	155	120.70	6.98	4.92	<0.0001	1.28	1.16	1.44
49	Triadic	535	153	121.00	6.98	4.59	<0.0001	1.26	1.14	1.42
50	Triadic	526	151	121.14	6.88	4.34	<0.0001	1.25	1.13	1.40
51	Triadic	516	147	120.92	6.85	3.81	<0.001	1.22	1.09	1.36
52	Triadic	504	142	120.23	6.84	3.19	0.001	1.18	1.06	1.33
53	Triadic	486	136	117.87	6.67	2.72	0.007	1.15	1.04	1.30
54	Triadic	483	138	119.00	6.74	2.82	0.005	1.16	1.04	1.30
55	Triadic	475	137	118.79	6.64	2.74	0.006	1.15	1.04	1.30
56	Triadic	467	137	118.62	6.65	2.76	0.006	1.15	1.04	1.29
57	Triadic	459	133	118.38	6.65	2.20	0.028	1.12	1.01	1.26

58	Triadic	455	131	119.01	6.75	1.78	0.076	1.10	0.99	1.24
59	Triadic	449	130	119.08	6.74	1.62	0.105	1.09	0.98	1.22
60	Triadic	441	129	118.48	6.61	1.59	0.111	1.09	0.98	1.22

Note. Latency refers to the number of seconds after a focal bite was emitted, Influence type refers to the type of contagion under consideration, N total refers to the number of bite events relevant for this specific latency, N observed refers to the observed number of cases in which at least one other individual took a bite after the focal bite event within the given latency, N expected refers to the average number of expected cases over 1,000 permutations of the observed time series, SD expected is the associated standard deviation, Z-score refers to the Z-score computed as (N observed - N expected) / SD expected, p is the associated p-value, IRR refers to the Incidence Rate Ratio, a measure of effect size computed as N observed / N expected, IRR 95%CI low and IRR 95%CI high refer to the lower and the higher end of the associated 95% Confidence Interval respectively. Statistical inference is based on pointwise, two-sided Z-tests using latency-specific null distributions generated from 1,000 permutations.

Table S9. Statistics for the contagion of eating at the dyadic level in the constructed triads.

Latency	Influence type	N total	N observed	N expected	SD expected	Z-score	p	IRR	IRR 95%CI low	IRR 95%CI high
1	Dyadic	1411	38	37.94	6.01	0.01	0.992	1.00	0.76	1.46
2	Dyadic	1409	84	74.42	7.89	1.21	0.225	1.13	0.92	1.42
3	Dyadic	1408	122	107.61	8.82	1.63	0.103	1.13	0.98	1.35
4	Dyadic	1403	157	137.36	9.30	2.11	0.035	1.14	1.01	1.32
5	Dyadic	1399	189	164.48	9.98	2.46	0.014	1.15	1.03	1.30
6	Dyadic	1393	218	189.18	10.60	2.72	0.007	1.15	1.04	1.29
7	Dyadic	1381	241	210.87	11.28	2.67	0.008	1.14	1.04	1.28
8	Dyadic	1375	266	231.38	11.58	2.99	0.003	1.15	1.05	1.27
9	Dyadic	1366	285	249.93	11.90	2.95	0.003	1.14	1.04	1.25
10	Dyadic	1361	307	267.27	12.12	3.28	0.001	1.15	1.06	1.26
11	Dyadic	1345	318	280.65	12.11	3.08	0.002	1.13	1.04	1.24
12	Dyadic	1327	324	291.84	11.98	2.69	0.007	1.11	1.03	1.20
13	Dyadic	1310	323	301.75	11.70	1.82	0.069	1.07	1.00	1.15
14	Dyadic	1283	330	308.00	11.67	1.89	0.059	1.07	1.00	1.15
15	Dyadic	1265	337	315.11	11.55	1.89	0.058	1.07	1.00	1.15
16	Dyadic	1245	340	320.67	11.57	1.67	0.095	1.06	0.99	1.14
17	Dyadic	1229	343	325.74	11.37	1.52	0.129	1.05	0.99	1.13

18	Dyadic	1211	349	329.51	11.53	1.69	0.091	1.06	0.99	1.14
19	Dyadic	1197	350	333.53	11.79	1.40	0.163	1.05	0.98	1.13
20	Dyadic	1178	343	335.16	11.86	0.66	0.509	1.02	0.95	1.10
21	Dyadic	1163	344	337.44	11.91	0.55	0.582	1.02	0.95	1.10
22	Dyadic	1143	341	337.68	11.81	0.28	0.778	1.01	0.95	1.09
23	Dyadic	1129	341	339.02	11.74	0.17	0.866	1.01	0.94	1.08
24	Dyadic	1104	337	336.27	11.70	0.06	0.950	1.00	0.94	1.08
25	Dyadic	1084	332	334.63	11.37	-0.23	0.817	0.99	0.93	1.06
26	Dyadic	1056	324	330.00	11.25	-0.53	0.594	0.98	0.92	1.05
27	Dyadic	1038	322	327.76	11.25	-0.51	0.609	0.98	0.92	1.05
28	Dyadic	1024	320	326.57	11.15	-0.59	0.556	0.98	0.92	1.05
29	Dyadic	1006	317	323.63	11.18	-0.59	0.553	0.98	0.91	1.05
30	Dyadic	996	314	322.97	11.05	-0.81	0.417	0.97	0.91	1.04
31	Dyadic	981	308	319.61	10.99	-1.06	0.291	0.96	0.90	1.04
32	Dyadic	965	306	316.47	11.05	-0.95	0.343	0.97	0.90	1.03
33	Dyadic	951	297	313.64	11.06	-1.51	0.132	0.95	0.89	1.02
34	Dyadic	935	295	309.90	10.92	-1.36	0.173	0.95	0.89	1.02
35	Dyadic	920	281	306.34	10.86	-2.33	0.020	0.92	0.86	0.98
36	Dyadic	905	276	302.74	10.87	-2.46	0.014	0.91	0.85	0.98
37	Dyadic	883	271	296.50	10.61	-2.40	0.016	0.91	0.85	0.98

38	Dyadic	865	264	291.46	10.67	-2.57	0.010	0.91	0.84	0.97
39	Dyadic	849	262	286.83	10.63	-2.34	0.019	0.91	0.85	0.98
40	Dyadic	828	252	280.45	10.50	-2.71	0.007	0.90	0.84	0.97
41	Dyadic	807	237	273.97	10.34	-3.57	<0.001	0.87	0.80	0.93
42	Dyadic	786	230	267.33	10.11	-3.69	<0.001	0.86	0.80	0.93
43	Dyadic	767	221	261.37	9.88	-4.09	<0.0001	0.85	0.79	0.91
44	Dyadic	748	216	255.23	9.81	-4.00	<0.0001	0.85	0.79	0.91
45	Dyadic	743	211	253.64	9.77	-4.37	<0.0001	0.83	0.78	0.90
46	Dyadic	731	208	249.65	9.63	-4.33	<0.0001	0.83	0.78	0.90
47	Dyadic	720	205	245.91	9.47	-4.32	<0.0001	0.83	0.78	0.90
48	Dyadic	713	202	243.55	9.33	-4.45	<0.0001	0.83	0.77	0.90
49	Dyadic	707	198	241.49	9.41	-4.62	<0.0001	0.82	0.76	0.88
50	Dyadic	703	196	239.97	9.49	-4.64	<0.0001	0.82	0.76	0.89
51	Dyadic	694	193	236.67	9.39	-4.65	<0.0001	0.82	0.76	0.88
52	Dyadic	680	188	231.60	9.39	-4.64	<0.0001	0.81	0.75	0.88
53	Dyadic	662	180	225.37	9.29	-4.88	<0.0001	0.80	0.74	0.87
54	Dyadic	654	180	222.31	9.28	-4.56	<0.0001	0.81	0.75	0.88
55	Dyadic	650	177	220.76	9.28	-4.72	<0.0001	0.80	0.74	0.87
56	Dyadic	643	175	218.03	9.36	-4.60	<0.0001	0.80	0.74	0.88
57	Dyadic	636	174	215.42	9.33	-4.44	<0.0001	0.81	0.74	0.88

58	Dyadic	623	172	210.56	9.31	-4.14	<0.0001	0.82	0.75	0.89
59	Dyadic	612	166	206.28	9.08	-4.44	<0.0001	0.80	0.74	0.88
60	Dyadic	601	158	202.09	9.00	-4.90	<0.0001	0.78	0.72	0.85

Note. Latency refers to the number of seconds after a focal bite was emitted, Influence type refers to the type of contagion under consideration, N total refers to the number of bite events relevant for this specific latency, N observed refers to the observed number of cases in which at least one other individual took a bite after the focal bite event within the given latency, N expected refers to the average number of expected cases over 1,000 permutations of the observed time series, SD expected is the associated standard deviation, Z-score refers to the Z-score computed as (N observed - N expected) / SD expected, p is the associated p-value, IRR refers to the Incidence Rate Ratio, a measure of effect size computed as N observed / N expected, IRR 95%CI low and IRR 95%CI high refer to the lower and the higher end of the associated 95% Confidence Interval respectively. Statistical inference is based on pointwise, two-sided Z-tests using latency-specific null distributions generated from 1,000 permutations.

Table S10. Statistics for the contagion of eating at the triadic level in the constructed triads.

Latency	Influence type	N total	N observed	N expected	SD expected	Z-score	p	IRR	IRR 95%CI low	IRR 95%CI high
1	Triadic	1411	0	0.28	0.54	-0.52	0.606	0.00	0.00	0.00
2	Triadic	1409	0	1.00	0.99	-1.00	0.315	0.00	0.00	0.00
3	Triadic	1408	3	2.20	1.49	0.53	0.593	1.36	0.60	>1,000
4	Triadic	1403	6	3.87	1.93	1.10	0.269	1.55	0.75	6.00
5	Triadic	1399	7	5.89	2.37	0.47	0.641	1.19	0.63	3.48
6	Triadic	1393	8	8.21	2.84	-0.07	0.941	0.97	0.57	2.65
7	Triadic	1381	15	10.83	3.25	1.28	0.200	1.39	0.87	2.99
8	Triadic	1375	19	13.52	3.62	1.51	0.130	1.41	0.89	2.70
9	Triadic	1366	26	16.47	4.00	2.38	0.017	1.58	1.06	2.85
10	Triadic	1361	31	19.72	4.37	2.58	0.010	1.57	1.09	2.75
11	Triadic	1345	35	22.92	4.50	2.68	0.007	1.53	1.10	2.45
12	Triadic	1327	37	26.31	4.80	2.23	0.026	1.41	1.04	2.15
13	Triadic	1310	44	29.64	5.04	2.85	0.004	1.48	1.11	2.21
14	Triadic	1283	46	32.76	5.36	2.47	0.014	1.40	1.04	2.01
15	Triadic	1265	48	36.22	5.61	2.10	0.036	1.33	1.02	1.86
16	Triadic	1245	49	39.50	5.79	1.64	0.101	1.24	0.96	1.70
17	Triadic	1229	55	42.84	6.08	2.00	0.045	1.28	0.99	1.74

18	Triadic	1211	56	46.01	6.25	1.60	0.110	1.22	0.95	1.64
19	Triadic	1197	58	49.42	6.52	1.32	0.188	1.17	0.92	1.56
20	Triadic	1178	60	52.61	6.67	1.11	0.268	1.14	0.90	1.49
21	Triadic	1163	63	55.84	6.79	1.05	0.292	1.13	0.90	1.46
22	Triadic	1143	65	58.76	6.88	0.91	0.365	1.11	0.89	1.42
23	Triadic	1129	68	61.93	7.07	0.86	0.391	1.10	0.89	1.39
24	Triadic	1104	64	64.48	7.10	-0.07	0.946	0.99	0.82	1.26
25	Triadic	1084	66	67.06	7.20	-0.15	0.883	0.98	0.81	1.24
26	Triadic	1056	69	69.07	7.14	-0.01	0.992	1.00	0.83	1.25
27	Triadic	1038	72	71.53	7.16	0.07	0.947	1.01	0.84	1.23
28	Triadic	1024	73	74.20	7.27	-0.16	0.869	0.98	0.82	1.22
29	Triadic	1006	75	76.47	7.34	-0.20	0.842	0.98	0.83	1.19
30	Triadic	996	77	79.18	7.37	-0.30	0.768	0.97	0.82	1.18
31	Triadic	981	76	81.88	7.06	-0.83	0.405	0.93	0.79	1.10
32	Triadic	965	79	83.98	7.06	-0.71	0.480	0.94	0.80	1.12
33	Triadic	951	83	86.18	7.21	-0.44	0.659	0.96	0.82	1.14
34	Triadic	935	80	88.14	7.27	-1.12	0.263	0.91	0.78	1.07
35	Triadic	920	87	90.04	7.31	-0.42	0.678	0.97	0.83	1.14
36	Triadic	905	88	91.66	7.33	-0.50	0.617	0.96	0.83	1.14
37	Triadic	883	88	92.55	7.35	-0.62	0.536	0.95	0.82	1.12

38	Triadic	865	88	93.78	7.35	-0.79	0.432	0.94	0.82	1.10
39	Triadic	849	89	94.95	7.27	-0.82	0.414	0.94	0.81	1.10
40	Triadic	828	87	95.54	7.24	-1.18	0.239	0.91	0.79	1.06
41	Triadic	807	88	95.90	7.18	-1.10	0.271	0.92	0.80	1.08
42	Triadic	786	85	96.22	7.14	-1.57	0.116	0.88	0.77	1.03
43	Triadic	767	85	96.54	7.14	-1.62	0.106	0.88	0.77	1.02
44	Triadic	748	82	96.79	7.08	-2.09	0.037	0.85	0.74	0.98
45	Triadic	743	84	98.79	7.17	-2.06	0.039	0.85	0.74	0.99
46	Triadic	731	82	99.87	7.27	-2.46	0.014	0.82	0.72	0.95
47	Triadic	720	83	100.78	7.19	-2.47	0.013	0.82	0.72	0.96
48	Triadic	713	84	102.10	7.24	-2.50	0.012	0.82	0.72	0.95
49	Triadic	707	85	103.60	7.30	-2.55	0.011	0.82	0.72	0.95
50	Triadic	703	86	105.42	7.45	-2.61	0.009	0.82	0.71	0.94
51	Triadic	694	86	106.39	7.43	-2.74	0.006	0.81	0.71	0.93
52	Triadic	680	84	106.40	7.41	-3.02	0.003	0.79	0.69	0.90
53	Triadic	662	85	105.69	7.35	-2.81	0.005	0.80	0.70	0.92
54	Triadic	654	85	106.57	7.35	-2.94	0.003	0.80	0.70	0.91
55	Triadic	650	88	107.94	7.43	-2.68	0.007	0.82	0.72	0.94
56	Triadic	643	88	108.81	7.37	-2.82	0.005	0.81	0.71	0.93
57	Triadic	636	90	109.57	7.41	-2.64	0.008	0.82	0.72	0.94

58	Triadic	623	89	109.33	7.38	-2.76	0.006	0.81	0.71	0.94
59	Triadic	612	91	109.40	7.49	-2.46	0.014	0.83	0.73	0.95
60	Triadic	601	94	109.33	7.41	-2.07	0.038	0.86	0.75	0.98

Note. Latency refers to the number of seconds after a focal bite was emitted, Influence type refers to the type of contagion under consideration, N total refers to the number of bite events relevant for this specific latency, N observed refers to the observed number of cases in which at least one other individual took a bite after the focal bite event within the given latency, N expected refers to the average number of expected cases over 1,000 permutations of the observed time series, SD expected is the associated standard deviation, Z-score refers to the Z-score computed as (N observed - N expected) / SD expected, p is the associated p-value, IRR refers to the Incidence Rate Ratio, a measure of effect size computed as N observed / N expected, IRR 95%CI low and IRR 95%CI high refer to the lower and the higher end of the associated 95% Confidence Interval respectively. Statistical inference is based on pointwise, two-sided Z-tests using latency-specific null distributions generated from 1,000 permutations.

Table S11. Statistics for the within-individual temporal dependencies of laughter in the observed triads.

Latency	Influence type	N total	N observed	N expected	SD expected	Z-score	p	IRR	IRR 95%CI low	IRR 95%CI high
1	Within-ind.	1031	0	7.29	2.70	-2.70	0.007	0.00	0.00	0.00
2	Within-ind.	1026	3	23.72	4.67	-4.43	<0.0001	0.13	0.09	0.20
3	Within-ind.	1007	17	40.07	5.85	-3.95	<0.0001	0.42	0.33	0.58
4	Within-ind.	984	32	54.63	6.98	-3.24	0.001	0.59	0.47	0.77
5	Within-ind.	963	38	66.92	7.71	-3.75	<0.001	0.57	0.46	0.74
6	Within-ind.	948	50	77.81	8.10	-3.43	<0.001	0.64	0.53	0.80
7	Within-ind.	926	50	86.84	8.57	-4.30	<0.0001	0.58	0.48	0.71
8	Within-ind.	905	49	94.34	8.73	-5.19	<0.0001	0.52	0.44	0.63
9	Within-ind.	889	56	101.12	9.30	-4.85	<0.0001	0.55	0.47	0.67
10	Within-ind.	868	59	106.41	9.53	-4.97	<0.0001	0.55	0.47	0.67
11	Within-ind.	850	62	110.92	9.71	-5.04	<0.0001	0.56	0.48	0.67
12	Within-ind.	830	64	114.38	9.54	-5.28	<0.0001	0.56	0.48	0.68
13	Within-ind.	808	67	116.72	9.73	-5.11	<0.0001	0.57	0.50	0.69
14	Within-ind.	784	68	117.98	9.64	-5.19	<0.0001	0.58	0.50	0.69
15	Within-ind.	772	72	120.32	9.71	-4.98	<0.0001	0.60	0.52	0.71
16	Within-ind.	766	78	123.28	9.82	-4.61	<0.0001	0.63	0.55	0.75
17	Within-ind.	760	82	125.72	9.91	-4.41	<0.0001	0.65	0.56	0.77

18	Within-ind.	743	78	126.07	9.69	-4.96	<0.0001	0.62	0.53	0.73
19	Within-ind.	730	80	126.84	9.73	-4.82	<0.0001	0.63	0.54	0.74
20	Within-ind.	721	84	127.84	9.65	-4.54	<0.0001	0.66	0.57	0.77
21	Within-ind.	707	81	127.71	9.53	-4.90	<0.0001	0.63	0.55	0.74
22	Within-ind.	696	84	127.84	9.54	-4.60	<0.0001	0.66	0.57	0.76
23	Within-ind.	683	83	127.58	9.64	-4.62	<0.0001	0.65	0.56	0.76
24	Within-ind.	669	84	126.89	9.58	-4.48	<0.0001	0.66	0.58	0.77
25	Within-ind.	653	80	125.48	9.45	-4.82	<0.0001	0.64	0.56	0.74
26	Within-ind.	639	81	124.26	9.30	-4.65	<0.0001	0.65	0.57	0.76
27	Within-ind.	631	79	124.15	9.27	-4.87	<0.0001	0.64	0.56	0.74
28	Within-ind.	622	79	123.68	9.18	-4.87	<0.0001	0.64	0.56	0.74
29	Within-ind.	608	81	121.97	9.15	-4.48	<0.0001	0.66	0.58	0.77
30	Within-ind.	596	81	120.55	9.18	-4.31	<0.0001	0.67	0.59	0.78
31	Within-ind.	585	77	118.59	8.97	-4.64	<0.0001	0.65	0.56	0.76
32	Within-ind.	572	77	116.82	8.82	-4.51	<0.0001	0.66	0.57	0.77
33	Within-ind.	563	73	115.66	8.68	-4.91	<0.0001	0.63	0.55	0.74
34	Within-ind.	552	72	114.07	8.62	-4.88	<0.0001	0.63	0.55	0.74
35	Within-ind.	539	73	112.09	8.59	-4.55	<0.0001	0.65	0.57	0.76
36	Within-ind.	533	72	111.39	8.61	-4.57	<0.0001	0.65	0.56	0.76
37	Within-ind.	524	74	110.04	8.62	-4.18	<0.0001	0.67	0.58	0.79

38	Within-ind.	513	70	108.21	8.58	-4.45	<0.0001	0.65	0.56	0.76
39	Within-ind.	508	72	107.66	8.54	-4.18	<0.0001	0.67	0.58	0.78
40	Within-ind.	501	71	106.53	8.52	-4.17	<0.0001	0.67	0.57	0.78
41	Within-ind.	496	71	105.87	8.52	-4.09	<0.0001	0.67	0.58	0.79
42	Within-ind.	485	70	103.83	8.53	-3.97	<0.0001	0.67	0.58	0.80
43	Within-ind.	479	68	102.93	8.50	-4.11	<0.0001	0.66	0.56	0.79
44	Within-ind.	472	68	101.71	8.48	-3.97	<0.0001	0.67	0.57	0.80
45	Within-ind.	470	69	101.44	8.54	-3.80	<0.001	0.68	0.58	0.82
46	Within-ind.	458	72	99.08	8.43	-3.21	0.001	0.73	0.62	0.88
47	Within-ind.	445	71	96.50	8.27	-3.08	0.002	0.74	0.63	0.88
48	Within-ind.	435	69	94.62	8.13	-3.15	0.002	0.73	0.62	0.88
49	Within-ind.	433	69	94.36	8.15	-3.11	0.002	0.73	0.62	0.89
50	Within-ind.	424	67	92.57	8.03	-3.18	0.001	0.72	0.62	0.88
51	Within-ind.	418	67	91.45	8.07	-3.03	0.002	0.73	0.62	0.89
52	Within-ind.	411	66	90.16	7.98	-3.03	0.002	0.73	0.62	0.89
53	Within-ind.	406	63	89.22	7.92	-3.31	<0.001	0.71	0.60	0.86
54	Within-ind.	401	65	88.25	7.84	-2.97	0.003	0.74	0.62	0.89
55	Within-ind.	399	68	88.02	7.86	-2.55	0.011	0.77	0.66	0.94
56	Within-ind.	391	63	86.28	7.72	-3.02	0.003	0.73	0.62	0.89
57	Within-ind.	384	62	84.88	7.60	-3.01	0.003	0.73	0.62	0.89

58	Within-ind.	377	61	83.41	7.59	-2.95	0.003	0.73	0.62	0.89
59	Within-ind.	373	61	82.63	7.57	-2.86	0.004	0.74	0.63	0.90
60	Within-ind.	369	62	81.82	7.50	-2.64	0.008	0.76	0.64	0.92

Note. Latency refers to the number of seconds after a focal laughter was emitted, Influence type refers to the type of contagion under consideration, N total refers to the number of laughter events relevant for this specific latency, N observed refers to the observed number of cases in which the individual laughed after the focal laughter within the given latency, N expected refers to the average number of expected cases over 1,000 permutations of the observed time series, SD expected is the associated standard deviation, Z-score refers to the Z-score computed as (N observed - N expected) / SD expected, p is the associated p-value, IRR refers to the Incidence Rate Ratio, a measure of effect size computed as N observed / N expected, IRR 95%CI low and IRR 95%CI high refer to the lower and the higher end of the associated 95% Confidence Interval respectively. Statistical inference is based on pointwise, two-sided Z-tests using latency-specific null distributions generated from 1,000 permutations.

Table S12. Statistics for the within-individual temporal dependencies of eating in the observed triads.

Latency	Influence type	N total	N observed	N expected	SD expected	Z-score	p	IRR	IRR 95%CI low	IRR 95%CI high
1	Within-ind.	1426	0	12.85	3.64	-3.53	<0.001	0.00	0.00	0.00
2	Within-ind.	1424	0	38.21	6.02	-6.35	<0.0001	0.00	0.00	0.00
3	Within-ind.	1418	4	61.56	7.29	-7.89	<0.0001	0.06	0.05	0.08
4	Within-ind.	1413	18	82.36	8.32	-7.73	<0.0001	0.22	0.18	0.27
5	Within-ind.	1408	25	100.57	9.34	-8.09	<0.0001	0.25	0.21	0.30
6	Within-ind.	1401	40	117.48	10.05	-7.71	<0.0001	0.34	0.29	0.40
7	Within-ind.	1388	49	132.23	10.67	-7.80	<0.0001	0.37	0.32	0.44
8	Within-ind.	1376	63	145.08	11.10	-7.40	<0.0001	0.43	0.38	0.51
9	Within-ind.	1360	74	156.05	11.39	-7.20	<0.0001	0.47	0.41	0.56
10	Within-ind.	1344	83	165.62	11.70	-7.06	<0.0001	0.50	0.44	0.58
11	Within-ind.	1319	107	172.67	12.01	-5.47	<0.0001	0.62	0.55	0.72
12	Within-ind.	1299	119	179.12	12.18	-4.94	<0.0001	0.66	0.59	0.77
13	Within-ind.	1281	126	184.94	12.06	-4.89	<0.0001	0.68	0.61	0.78
14	Within-ind.	1249	137	187.73	12.08	-4.20	<0.0001	0.73	0.65	0.84
15	Within-ind.	1216	145	189.53	12.17	-3.66	<0.001	0.77	0.68	0.88
16	Within-ind.	1178	151	189.65	12.10	-3.19	0.001	0.80	0.71	0.91
17	Within-ind.	1156	157	191.26	12.24	-2.80	0.005	0.82	0.73	0.94

18	Within-ind.	1130	166	191.73	12.29	-2.09	0.036	0.87	0.77	0.99
19	Within-ind.	1106	171	192.00	12.31	-1.71	0.088	0.89	0.79	1.02
20	Within-ind.	1073	175	190.10	12.34	-1.22	0.221	0.92	0.82	1.05
21	Within-ind.	1047	174	188.93	12.20	-1.22	0.221	0.92	0.82	1.06
22	Within-ind.	1021	174	187.25	11.97	-1.11	0.269	0.93	0.83	1.06
23	Within-ind.	994	168	185.15	11.65	-1.47	0.141	0.91	0.81	1.04
24	Within-ind.	969	169	182.91	11.58	-1.20	0.230	0.92	0.82	1.05
25	Within-ind.	937	161	178.96	11.21	-1.60	0.109	0.90	0.80	1.02
26	Within-ind.	903	154	174.35	11.02	-1.85	0.065	0.88	0.79	1.01
27	Within-ind.	882	156	172.06	10.96	-1.47	0.143	0.91	0.81	1.04
28	Within-ind.	866	159	170.62	10.86	-1.07	0.285	0.93	0.83	1.07
29	Within-ind.	846	159	168.17	10.69	-0.86	0.391	0.95	0.84	1.08
30	Within-ind.	824	150	164.99	10.50	-1.43	0.153	0.91	0.81	1.04
31	Within-ind.	804	149	162.77	10.44	-1.32	0.187	0.92	0.81	1.05
32	Within-ind.	793	147	161.50	10.39	-1.40	0.163	0.91	0.81	1.04
33	Within-ind.	773	142	158.30	10.14	-1.61	0.108	0.90	0.80	1.02
34	Within-ind.	748	135	153.95	9.84	-1.92	0.054	0.88	0.78	1.00
35	Within-ind.	730	134	150.83	9.78	-1.72	0.085	0.89	0.79	1.02
36	Within-ind.	720	136	149.38	9.77	-1.37	0.171	0.91	0.81	1.05
37	Within-ind.	702	134	146.24	9.73	-1.26	0.208	0.92	0.81	1.05

38	Within-ind.	681	127	142.30	9.46	-1.62	0.106	0.89	0.79	1.02
39	Within-ind.	668	127	140.07	9.43	-1.39	0.166	0.91	0.80	1.04
40	Within-ind.	651	125	136.74	9.37	-1.25	0.210	0.91	0.80	1.06
41	Within-ind.	628	120	132.25	9.22	-1.33	0.184	0.91	0.80	1.05
42	Within-ind.	607	120	128.01	8.94	-0.90	0.370	0.94	0.82	1.08
43	Within-ind.	588	120	124.27	8.74	-0.49	0.625	0.97	0.84	1.12
44	Within-ind.	573	120	121.34	8.60	-0.16	0.876	0.99	0.87	1.15
45	Within-ind.	567	121	120.20	8.53	0.09	0.925	1.01	0.88	1.17
46	Within-ind.	559	120	118.68	8.58	0.15	0.878	1.01	0.89	1.18
47	Within-ind.	553	118	117.57	8.52	0.05	0.960	1.00	0.88	1.17
48	Within-ind.	544	116	115.82	8.42	0.02	0.983	1.00	0.88	1.17
49	Within-ind.	535	114	113.92	8.33	0.01	0.992	1.00	0.88	1.17
50	Within-ind.	526	110	112.03	8.23	-0.25	0.805	0.98	0.86	1.14
51	Within-ind.	516	108	110.01	8.16	-0.25	0.805	0.98	0.86	1.15
52	Within-ind.	504	107	107.65	7.97	-0.08	0.935	0.99	0.87	1.16
53	Within-ind.	486	102	103.87	7.77	-0.24	0.810	0.98	0.85	1.15
54	Within-ind.	483	103	103.28	7.75	-0.04	0.971	1.00	0.87	1.17
55	Within-ind.	475	97	101.57	7.72	-0.59	0.554	0.96	0.83	1.14
56	Within-ind.	467	93	99.88	7.72	-0.89	0.373	0.93	0.81	1.11
57	Within-ind.	459	91	98.15	7.74	-0.92	0.355	0.93	0.80	1.10

58	Within-ind.	455	93	97.32	7.73	-0.56	0.577	0.96	0.83	1.14
59	Within-ind.	449	93	96.03	7.67	-0.40	0.692	0.97	0.84	1.16
60	Within-ind.	441	91	94.30	7.57	-0.44	0.663	0.96	0.84	1.15

Note. Latency refers to the number of seconds after a focal bite was emitted, Influence type refers to the type of contagion under consideration, N total refers to the number of bite events relevant for this specific latency, N observed refers to the observed number of cases in which the individual took a bite after the focal bite event within the given latency, N expected refers to the average number of expected cases over 1,000 permutations of the observed time series, SD expected is the associated standard deviation, Z-score refers to the Z-score computed as (N observed - N expected) / SD expected, p is the associated p-value, IRR refers to the Incidence Rate Ratio, a measure of effect size computed as N observed / N expected, IRR 95%CI low and IRR 95%CI high refer to the lower and the higher end of the associated 95% Confidence Interval respectively Statistical inference is based on pointwise, two-sided Z-tests using latency-specific null distributions generated from 1,000 permutations.

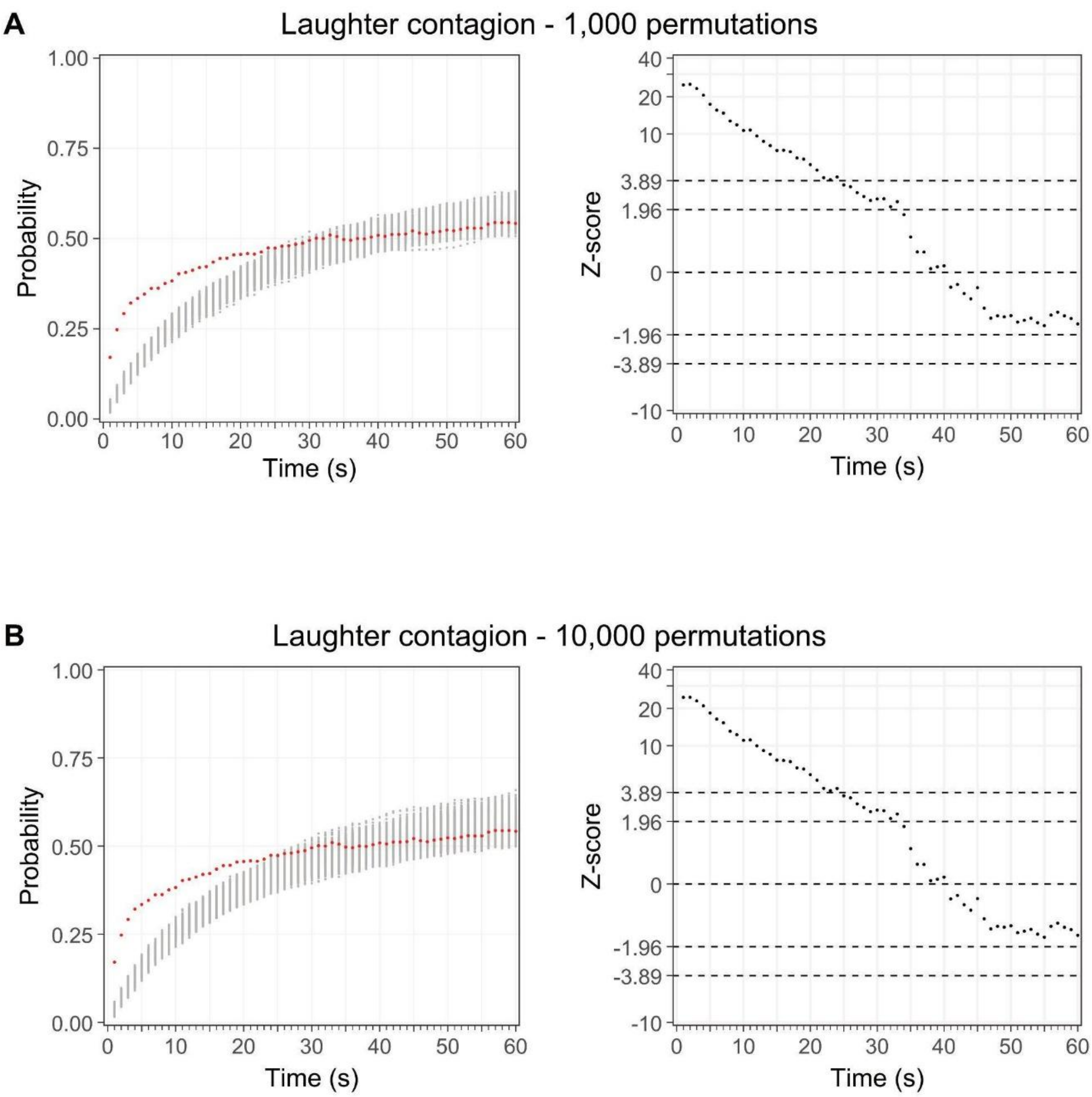

Supplementary Figure 2. **Effect of the number of permutations on the inference of the behavioural contagion of laughter.** A. Behavioural contagion inference derived from 1,000 permutations (identical to Figure 3B in the main text). B. Behavioural contagion inference derived from 10,000 permutations (note that the density of grey dots is higher in the B left panel compared to the A left panel). The inferences derived from both permutation numbers are nearly identical (compare A and B right panels). On all left panels, $|Z| \geq 1.96$ indicates $p \leq 0.05$ and $|Z| \geq 3.89$ indicates $p \leq 0.0001$. N = 1,031 laughter events; one event was excluded because it occurred within 1 s of the end of the interaction and could therefore not be evaluated for potential contagion. Statistical inference is based on pointwise, two-sided Z-tests using permutation-derived null distributions generated from 1,000 permutations in A and from 10,000 permutations in B.

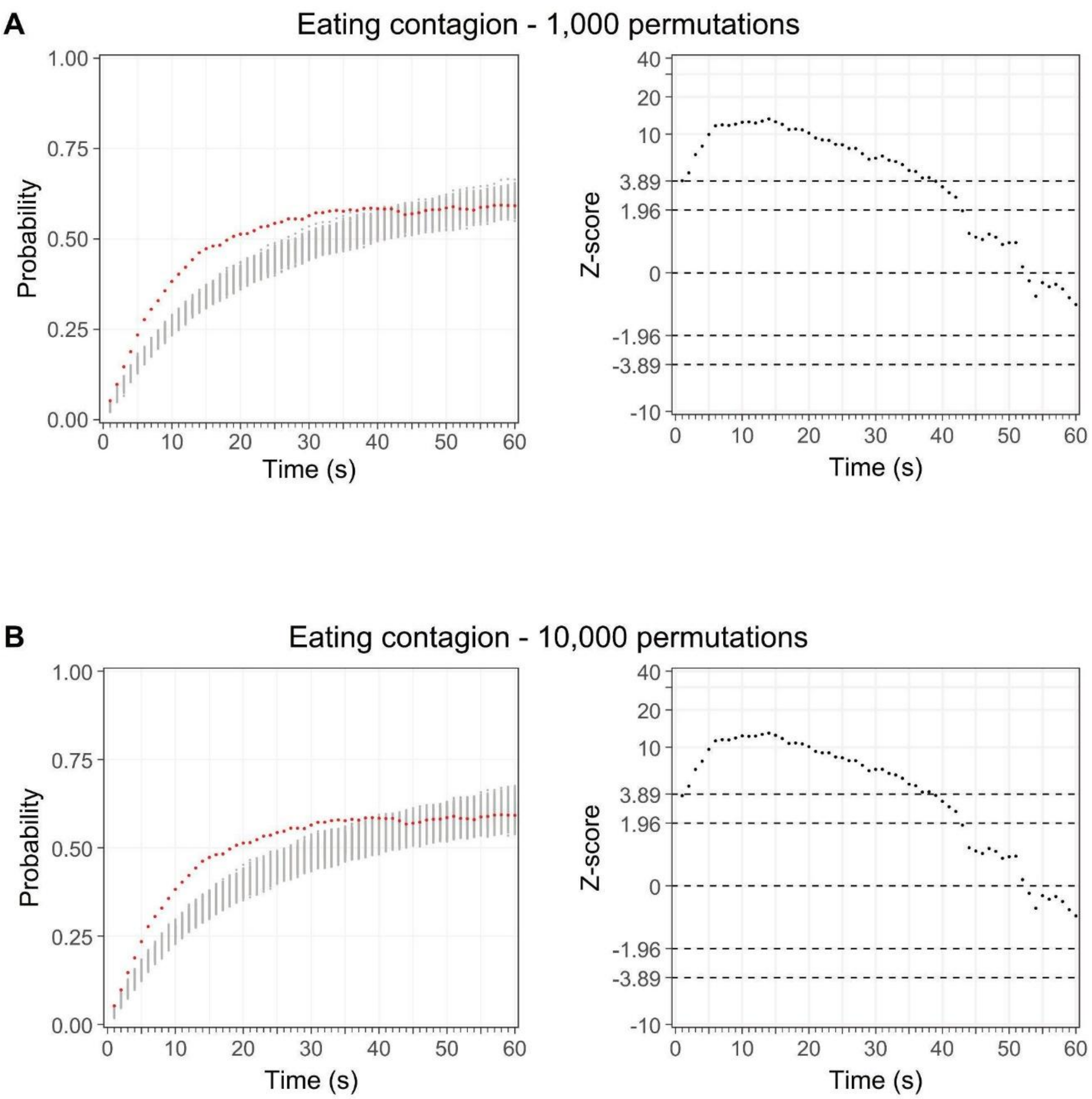

Supplementary Figure 3. **Effect of the number of permutations on the inference of the behavioural contagion of eating.** A. Behavioural contagion inference derived from 1,000 permutations (identical to Figure 5B in the main text). B. Behavioural contagion inference derived from 10,000 permutations (note that the density of grey dots is higher in the B left panel compared to the A left panel). The inferences derived from both permutation numbers are nearly identical (compare A and B right panels). On all left panels, $|Z| \geq 1.96$ indicates $p \leq 0.05$ and $|Z| \geq 3.89$ indicates $p \leq 0.0001$. N = 1,426 bite events; one event was excluded because it occurred within 1 s of the end of the interaction and could therefore not be evaluated for potential contagion. Statistical inference is based on pointwise, two-sided Z-tests using permutation-derived null distributions generated from 1,000 permutations in A and from 10,000 permutations in B.
